# Modified Si Miao Powder granules alleviates osteoarthritis progression by regulating M1/M2 polarization of macrophage through NF-κB signaling pathway

**DOI:** 10.3389/fphar.2024.1361561

**Published:** 2024-06-21

**Authors:** Qi He, Ding Tian, Zhiyuan Wang, Dan Zheng, Liqiang Zhi, Jianbing Ma, Jing An, Rui Zhang

**Affiliations:** ^1^ Translational Medicine Center, Honghui Hospital, Xi’an Jiaotong University, Xi’an, Shaanxi, China; ^2^ Department of Joint Surgery, Honghui Hospital, Xi’an Jiaotong University, Xi’an, Shaanxi, China; ^3^ Department of Medical Technology, Guiyang Healthcare Vocational University, Guiyang, Guizhou, China

**Keywords:** osteoarthritis, inflammation, modified Si Miao Powder, macrophage polarization, NF-κB signaling pathway

## Abstract

**Background:**

Osteoarthritis (OA) is a chronic degenerative disease mainly characterized by cartilage damage and synovial inflammation. Si Miao Powder, an herbal formula, was recorded in ancient Chinese medicine prescription with excellent anti-inflammatory properties. Based on the classical formula, the modified Si Miao Powder (MSMP) was developed with the addition of two commonly Chinese orthopedic herbs, which had the efficacy of strengthening the therapeutic effect for OA.

**Methods:**

In the *in vivo* experiments, thirty-six 8-week-old male C57BL/6 mice were randomly divided into six groups: sham group, OA group, celecoxib group, low-MSMP group, middle-MSMP group, and high-MSMP group. OA mice were constructed by destabilization of medial meniscus (DMM) and treated with MSMP granules or celecoxib by gavage. The effects of MSMP on cartilage, synovitis and inflammatory factor of serum were tested. For *in vitro* experiments, control serum and MSMP-containing serum were prepared from twenty-five C57BL/6 mice. Macrophages (RAW264.7 cells) were induced by lipopolysaccharide (LPS) and then treated with MSMP-containing serum. The expression of inflammatory factors and the change of the NF-κB pathway were tested.

**Results:**

*In vivo*, celecoxib and MSMP alleviated OA progression in the treated groups compared with OA group. The damage was partly recovered in cartilage, the synovial inflammatory were reduced in synovium, and the concentrations of IL-6 and TNF-α were reduced and the expression of IL-10 was increased in serum. The function of the middle MSMP was most effective for OA treatment. The results of *in vitro* experiments showed that compared with the LPS group, the MSMP-containing serum significantly reduced the expression levels of pro-inflammatory (M1-type) factors, such as CD86, iNOS, TNF-α and IL-6, and promoted the expression levels of anti-inflammatory (M2-type) factors, such as Arg1 and IL-10. The MSMP-containing serum further inhibited NF-κB signaling pathway after LPS induction.

**Conclusion:**

The study demonstrated that MSMP alleviated OA progression in mice and MSMP-containing serum modulated macrophage M1/M2 phenotype by inhibiting the NF-κB signaling pathway. Our study provided experimental evidence and therapeutic targets of MSMP for OA treatment.

## 1 Introduction

Osteoarthritis (OA) is a multifactorial degenerative joint disease that is highly prevalent in the elderly population. OA is mainly characterized by the degeneration of articular cartilage, often accompanied by synovial inflammation. The clinical manifestations of the disease involve in recurrent joint swelling, pain, stiffness, and deformity in late stages. The 2019 Evidence-Based Guidelines for OA, developed by the American College of Rheumatology in collaboration with the Arthritis Foundation, indicate that approximately 302 million people worldwide suffered from OA disease (Kolasinski et al., 2020). The non-steroidal anti-inflammatory drugs are the first-line drugs for the treatment of OA in clinic. Celecoxib, is widely used in clinical practice for OA treatment and can effectively alleviate the symptoms of arthritis. However, OA is a chronic disease, and long-term or intermittent repeated use of non-steroidal anti-inflammatory drugs will cause varying degrees of damage to the liver, kidney and gastrointestinal tract of the elderly patients. OA can be treated by arthroplasty in the late stage, but due to the shortcomings of joint replacement, such as major surgical trauma, high surgical costs and a limited lifespan, there exist limitations for the surgical operation. Therefore, the search for natural herbal drugs that can effectively treat OA has become a focus in pharmacological research (Li and Zhang, 2020). The active ingredients in many botanical drugs have been shown to have anti-inflammatory and analgesic effects (Chen et al., 2016). Chinese herbal medicines or formulas are gradually being used as one of the methods to combat OA in clinical practice, and the use of botanical drugs in the early and intermediate stages of OA may reduce the rate of artificial joint replacement surgery for patients (Cho et al., 2021).

OA is classified as a “bone bi disease” in Chinese medicine, which is believed to develop when the bone marrow of the body is empty and the external evil enters the body mainly due to the feeling of wind, dampness, heat and cold, thereby resulting in unfavorable bone joints (Zhang et al., 2023). The classic formula Si Miao Powder, recorded in the book *Singing Convenience Reading* by a doctor called Zhang Bingcheng in the Qing Dynasty, is a typical formula for the treatment of impotence caused by dampness-heat downstream injection. This formula has the effect of clearing away heat and eliminating dampness, and consists of four herbs: Atractylodes Lancea (Thunb.) Dc. [Asteraceae; Atractylodes Rhizome], Phellodendron amurense Rupr. [Rutaceae; Cortex phellodendri], Achyranthes bidentata Blume [Amaranthaceae; Achyranthis Bidentatae Radix] and Coix lacryma-jobi var. ma-yuen (Rom.Caill.) Stapf [Poaceae; Coicis Semen]. In recent years, Si Miao Powder has been widely used in clinical practice for arthritis treatment. For example, Peng et al. found that Si Miao Powder was effective in the treatment of rheumatoid arthritis (Peng et al., 2021). Recently, Cao et al. reported that Si Miao Powder improved the serum uric acid level and promoted the activation of M2-type macrophages in hyperuricemic mice (Cao et al., 2022).

According to the therapeutic principle of “treating the underlying cause before treating the symptoms” and “treating both the symptoms and the underlying cause” in Chinese medicine compound prescription, Dipsacus asper Wall.ex DC [Dipsacaceae; Dipsaci Radix] and Drynaria roosii Nakaike [Polypodiaceae; Drynariae Rhizoma] are added in the formula of Si Miao Powder in the present study, called as Modified Si Miao Powder (MSMP). Based on the basic theory of traditional Chinese medicine that “the kidney governing bones,” the addition of Dipsaci Radix and Drynariae Rhizoma has the effects of tonifying the liver and kidneys, and strengthening the tendons and bones. In traditional Chinese medicine, Dipsaci Radix and Drynariae Rhizoma are often applied in combination as a drug pair to aid the treatment of osteoporosis, osteoarthritis, and other orthopedic disorders. In the current study, the *in vivo* experiment was conducted to investigate the therapeutic effect of MSMP on OA progression, and the *in vitro* experiment was implemented to explore the function of MSMP-containing serum on the polarization of synovial macrophages and the underlying molecular mechanism.

## 2 Materials and methods

### 2.1 Preparation of MSMP

The Chinese medicine formula of MSMP includes Atractylodes Rhizome, Cortex phellodendri, Achyranthis Bidentatae Radix, Coicis Semen, Dipsaci Radix and Drynariae Rhizoma. The six ingredients of MSMP are granules and sourced from Pharmacy Department of Xi’an Honghui Hospital. The daily dose of MSMP for adults is 32.5 g, which is equivalent to 85 g herbs. The information of MSMP granules including drug composition, dosage system, batch numbers, and record numbers, was shown in Table 1. An MSMP dose of 17 g/kg was identified as a middle dosage to use in animal gavage according to the conversion standard of human-animal drug dose (Nair and Jacob, 2016). The MSMP granules were dissolved in double-distilled water (ddH_2_O) for the *in vivo* experiments.

**TABLE 1:** The information of MSMP formula.

Scientific name of the herb	Chinese name	Material	Dosage (g)^a^	Batch No.	Record No.
*Atractylodes Lancea (Thunb.) DC.*	Cang Zhu	Rootstalk	15	22045681	1121000040000
*Phellodendron amurense Rupr.*	Huang Bai	Bark	10	23032061	1121000120000
*Achyranthes bidentata Blume*	Niu Xi	Root	15	23000831	1121000212000
*Coix lacryma-jobi var. ma-yuen (Rom.Caill.) Stapf*	Yi Yi Ren	Kernel	15	23012191	1121000068000
*Dipsacus asper Wall.ex DC.*	Xu Duan	Root	15	23025771	1121000199000
*Drynaria roosii Nakaike*	Gu Sui Bu	Rootstalk	15	22043861	1121000061000

^a^ Dry weight of the medicinal substance.

The plant name has been in accordance with http://www.plantsoftheworldonline.org.

### 2.2 Ultra-high-pressure liquid chromatography (UHPLC-MS/MS) analysis of MSMP sample

The analysis method for chromatography-mass spectrometry was supplied by Sci-Tech Innovation Co., Ltd. (Qingdao, China). The first step was sample detection. The well-mixed sample (100 mg) of was placed in a 2 mL centrifuge tube, added 1 mL of 70% methanol and 3 mm steel beads to vibrate and pulverize with an automatic sample rapid grinder (JXFSTPRP-48, 70 Hz), and crushed with low temperature ultrasound (40 kHz). Nex, the sample was centrifuged at 12,000 rpm at 4°C and the supernatant was diluted 100 times. Then the 10 μL of 100 μg/mL internal standard was added and passed through a PTFE filter with a thickness of 0.22 μm. The second step was quantitative analysis of metabolites. The relative concentration and percent of metabolites in the sample were calculated based on the peak area. The third step was chromatography-mass spectrometry analysis. The LC-MS (Ultimate 3000 LC, Q Exactive HF) analysis platform and C18 chromatography column (Zorbax Eclipse C18 (1.8 μm × 2.1 mm × 100 mm) was used for detection.

### 2.3 Animals and reagents

Sixty-one 8-week-old C57BL/6 mice (Experimental Animal Center of Xi’an Jiaotong University) weighing 20–22 g were kept in a standard environment with suitable temperature and humidity and with food and water *ad libitum*. Thirty-six mice were used for *in vivo* experiments while twenty-five mice were used for *in vitro* experiments. All mice were conducted following the Guidelines for the Care and Use of Animals in the Laboratory of the National Institutes of Health (Kastenmayer et al., 2012). The animal study had the approval of the Experimental Animal Center of Xi’an Jiaotong University. The reagents in the current study were as follows: Lipopolysaccharide (Sigma), p65 Antibody (Abways, CY5034), p-p65 Antibody (Abways, CY6372), β-actin Antibody (Boster, BM3873), Col2a1 Antibody (Boster, BA0533), Mmp13 Antibody (Boster, BA2204), iNOS Antibody (Boster, BA0362), Arg1 Antibody (Servicebio, GB11285-100), IL-6 ELISA Kit (Boster), TNF-α ELISA Kit (Boster), IL-10 ELISA Kit (RuiXin Bio), HE Staining Kit (Solarbio), Senna O Solid Green Staining Kit (Solarbio) Animal Surgical Instruments, Absorbable Surgical Suture (6-0), Gavage Needle (12 gauge), Tissue Decalcification Solution (Bosterbio), Tissue Fixation Solution (Bosterbio), and Sodium Pentobarbital.

### 2.4 Experimental OA mouse model and treatment

The animal model of OA was established by destabilization of the medial meniscus (DMM) in the right knee joints of C57BL/6 mice. Briefly, under sterile conditions, the mouse was anesthetized by intraperitoneal injection of 0.3% pentobarbital sodium (30 mg/kg) and the patellar ligament was exposed. Then the fat under the patellar ligament was bluntly dissected with a scalpel along the medial patellar ligament for about 2 mm. Subsequently, the medial meniscotibial ligament was transected so that the meniscus was in an unstable state, and finally the wound was sutured. Thirty-six C57BL/6 mice were randomly divided into six groups (*n* = 6): sham group, OA group, celecoxib group, low-MSMP group, middle-MSMP group, and high-MSMP group. Except for the sham group, mice in all groups were subjected to the surgery of DMM. The ratio of low, middle and high dosages for treatment was 1:2:4 (the amount of raw drug was 8.5 g/kg, 17 g/kg and 34 g/kg respectively). Celecoxib Capsules (specification: 0.2 g/capsule, manufacturer: Sichuan Gowei Pharmaceutical Co., Ltd., record no.: H20203357, batch no.: 20220218) are sourced from Pharmacy Department of Xi’an Honghui Hospital. The celecoxib group was given celecoxib solution (20 mg/kg). Three weeks after surgery, the mice were treated by gavage. The treated drug for each group was administered once a day at a fixed time for six consecutive weeks. Meanwhile, an equal quantity of ddH_2_O was given to the sham group and OA group.

### 2.5 Histological staining and analysis

Six weeks after gavage treatment, the right knee joints of the mice were collected and fixed in 4% paraformaldehyde for 48 h. Then the joints were decalcified for 3 weeks, subjected to paraffin embedding and sectioned at a thickness of 5 μm. The paraffin sections were stained with hematoxylin and eosin (HE) and Safranin O and fast green respectively and sealed by resin for observation and photography under light microscope. The cartilage degeneration and the severity of knee OA were evaluated by the Osteoarthritis Research Society International (OARSI) scoring criteria ranging from 0 to 24 (Pritzker et al., 2006). The histopathologic grading of inflammation in synovium was performed according to the synovial score (Krenn et al., 2006).

### 2.6 Immunohistochemistry

The paraffin sections were deparaffinized and rehydrated in xylene. Subsequently, 3% H_2_O_2_ was added to block endogenous peroxidase activity. Then, the antigen was digested with 0.1% trypsin and blocked in goat serum. The sections were then sequentially incubated with primary antibody overnight at 4°C and secondary antibody for 1 h at 37°C. Finally, horseradish peroxidase-conjugated streptavidin-biotin was added and immunoreactivity was visualized under a light microscope.

### 2.7 Enzyme-linked immunosorbent assay (ELISA)

The blood in each group was collected and left in the EP tubes for 30 min, then centrifuged with 3,000 g for 20 min at 4°C to collect the serum and stored at −80°C until use. Each sample was tested in triplicate. The expression levels of IL-6, TNF-α, and IL-10 were detected using ELISA kits according to the instructions of the manufactures.

### 2.8 Preparation of control and drug-containing serum

Twenty-five mice were randomly divided into five groups to collect serum. The mice of three groups were gavaged by low, middle and high dosage MSMP to collect MSMP-containing serum, and the mice of the other two groups were gavaged with the same volume of ddH_2_O to collect control serum. One week later, the serum samples were collected and inactivated at 56°C for 30 min, then filtered through a 0.22 μm filtration membrane to remove bacteria. The serum was stored at −80°C for next experiments.

### 2.9 Cell culture and treatment

RAW264.7 macrophages were purchased from the Stem Cell Bank of the Chinese Academy of Sciences (Shanghai, China). The cells were cultured in DMEM high-sugar medium supplemented with 10% fetal bovine serum (Gibco, Australia), 100 U/mL penicillin, and 0.1 mg/mL streptomycin (Macgene, Beijing, China), and incubated in an incubator at 37°C with humidified 5% CO_2._ When the cells reached a confluence of 70%, LPS (1 μg/mL) was added to induce the cells. 24 h later, the control and LPS groups were treated with the control serum, and the low-, middle- and high-MSMP groups were treated with the corresponding MSMP-containing serum.

### 2.10 RNA extraction and real-time quantitative PCR (RT-qPCR)

Macrophages were seeded into 6-well plates (5 × 10^5^/well) and the macrophages of MSMP groups were incubated with 10% MSMP-containing serum for 24 h post LPS (1 μg/mL) induction. The total RNA was extracted from the cells using the SPARKeasy Cell RNA Kit. The RNA concentration was determined using NanoDrop 2000. Subsequently, the RNA was reverse-transcribed into a stable cDNA using the SPARKscript 1st Strand cDNA Synthesis Kit (with gDNA Eraser). The mRNA expression of CD86, iNOS, TNF-α, IL-6, Arg1, IL-10, and β-actin was tested. The primer information was shown in Table 2. The reaction of qPCR was performed on a qPCR system (Bio-Rad Lab, United States) under the following conditions: 10 min at 95°C followed by 40 cycles of 95°C for 15 s and 58°C for 60 s. The relative expression levels of target genes were normalized to β-actin and analyzed using the comparative 2^−ΔΔCt^ method.

**TABLE 2:** The primers for qPCR.

Genes	Forward (5′-3′)	Reverse (5′-3′)
CD86	TGT​CTG​ATC​TTG​CTA​GGA​CCG	GAG​AGT​AAC​GGC​CTT​TTT​GTG​A
iNOS	GTT​CTC​AGC​CCA​ACA​ATA​CAA​GA	GTG​GAC​GGG​TCG​ATG​TCA​C
TNF-a	CGATGGGTTGTACCTT	TACTTGGGCAGATTGAC
IL-6	AAT​TCG​GTA​CAT​CCT​CGC​GG	GGT​TGT​TTT​CTG​CCA​GTG​CC
Arg1	CTC​CAA​GCC​AAA​GTC​CTT​AGA​G	GGA​GCT​GTC​ATT​AGG​GAC​ATC​A
IL-10	CGG​GAA​GAC​AAT​AAC​TGC​ACC​C	CGG​TTA​GCA​GTA​TGT​TGT​CCA​GC
β-actin	CAT​TGC​TGA​CAG​GAT​GCA​GAA​GG	TGC​TGG​AAG​GTG​GAC​AGT​GAG​G

### 2.11 Western blotting analysis

Macrophages were seeded into 6-well plates (5 × 10^5^/well) and incubated with 10% middle-MSMP containing serum for 24 h after LPS (1 μg/mL) induction. The cells were lysed with RIPA lysate and 0. 1% PMSF. After centrifugation at 12,000 g for 10 min, the supernatants were collected and the protein concentration was measured using the BCA protein quantification kit. Then the protein was separated by the 10% dodecyl sulfate-polyacrylamide gel electrophoresis (SDS-PAGE), and transferred to a 0.45 μm polyvinylidene fluoride (PVDF) membrane. Non-specific proteins were blocked with 5% skimmed milk for 1 h at room temperature, and then the membranes were incubated with primary proteins such as β-actin (1:5,000), p-p65 (1:1,000) and p65 (1:5,000) at 4°C overnight. After being washed three times with TBST buffer solution, the membranes were incubated with horseradish peroxidase-conjugated anti-rabbit secondary antibodies for 1 h at room temperature, and washed three times again with TBST. The chemiluminescence signals of the bands were detected with Image Lab using the enhanced luminescence solution, and the expression intensity of protein blots was analyzed using ImageJ software.

### 2.12 Immunofluorescence assay

Macrophages were seeded on cell crawls in 12-well plates (1 × 10^5^/well), incubated with 10% middle-MSMP containing serum for 24 h after LPS (1 μg/mL) induction. Then the cells in the cell crawls were washed with PBS and fixed by 4% paraformaldehyde for 30 min. Subsequently, the cells were permeabilized with 0.3% Triton 100 × for 15 min, fixed with 3% BSA for 30 min and incubated with primary antibody p-p65 (1:200) at 4°C overnight. The next day, they were incubated with fluorescence labeled secondary antibody (1:1,000) at room temperature away from light for 1 h, stained by DAPI for 5 min, and finally blocked by glycerol. The images were acquired and analyzed with the fluorescence microscope (Leica).

### 2.13 Statistical analysis

All experiments in this study were replicated at least three times, and all data were statistically analyzed using GraphPad Prism eight software. Data were expressed as mean ± standard deviation (SD), differences between groups were analyzed via one-way analysis of variance (ANOVA), and *post hoc* tests were performed using Tukey’s multiple comparison test. *p*-value ≤ 0.05 were considered as statistically significant.

## 3 Results

### 3.1 The chemical compositions of MSMP

The chemical composition of MSMP was detected by UHPLC-MS/MS technology. The high-resolution molecular weight and MS/MS fragment information were obtained. The total ion chromatogram of MSMP granules was presented in positive and negative mode (Supplementary Figure S1). There were 30 main chemical components found in the MSMP (Supplementary Table S1). Alkaloids and flavonoids were abundant in the metabolites of MSMP.

### 3.2 MSMP inhibited articular cartilage damage in OA mice

**FIGURE 1:**
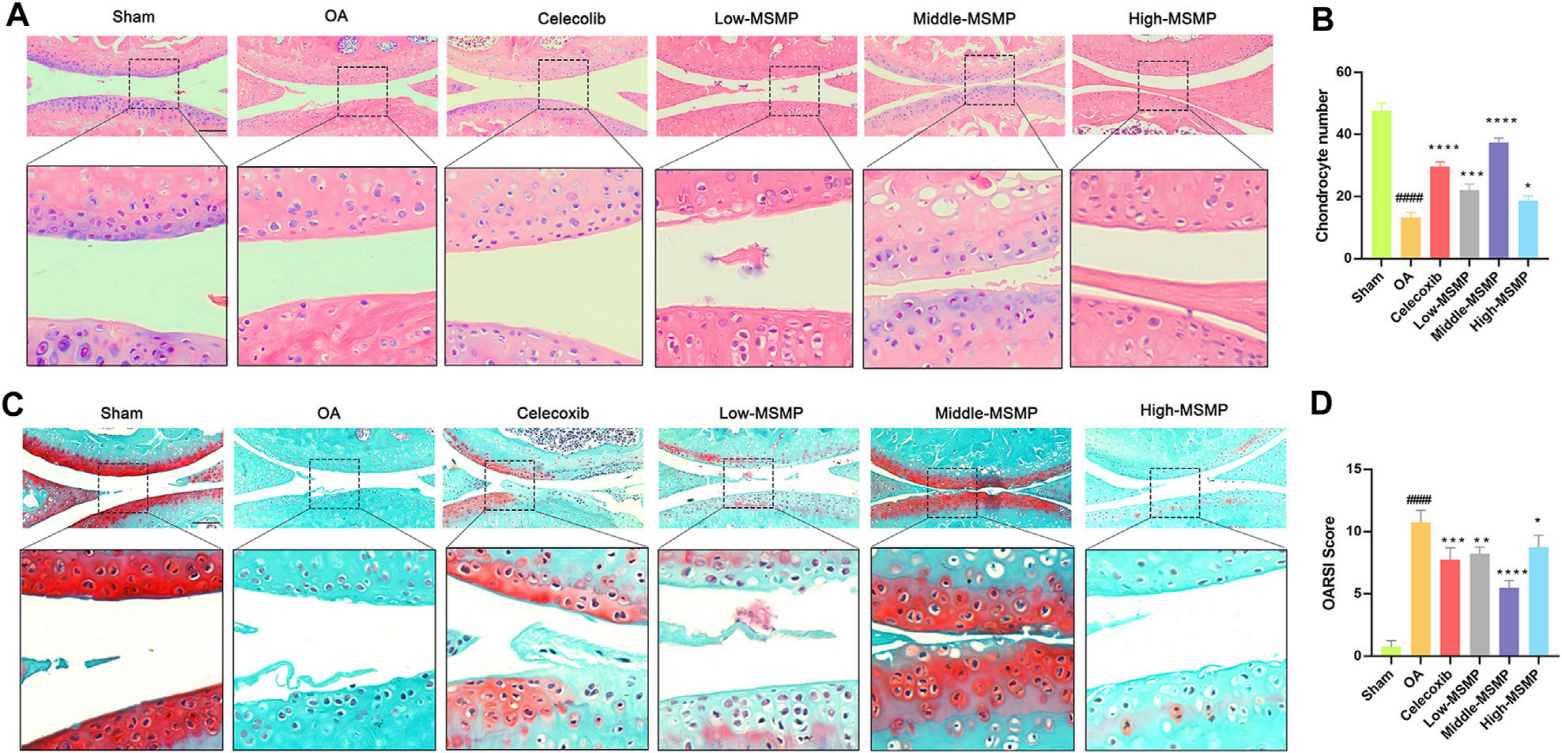
MSMP inhibited articular cartilage damage in OA mice. **(A)** HE staining of the articular cartilage tissues in mice. **(B)** Statistical analysis of the number of chondrocytes in each of the group. **(C)** Safranin O and fast green staining of the articular cartilage tissues in mice. **(D)** OARSI analysis in mice. Scale bar represents 100 μm (Date are mean ± SD, ^####^ *p* < 0.0001, versus Sham group; *p < 0.05, **p < 0.01, ***p < 0.001, ****p < 0.0001, versus OA group).

After 6 weeks of gavage treatment with celecoxib or different concentrations of MSMP, the therapeutic effects of MSMP on the damaged cartilage tissues of OA mice were tested by HE staining and Safranin O and fast green staining, and further evaluated by OARSI score system. The articular cartilage surface was smooth and structurally intact in the sham group, and there were obvious defects in the cartilage layer and reduction of chondrocytes in the OA group compared with the sham group. In the meantime, there were different degrees of improvement in the celecoxib group and in the MSMP-treated group compared with the OA group (Figures 1A, C). According to the statistical data and the evaluation results, the number of the chondrocytes in the articular cartilage was partly recovered and the OARSI score was partly reversed in the treated groups compared with the OA group (Figures 1B, D). The therapeutic effect in the middle-MSMP group exhibited the most increased trend in chondrocyte numbers and the most decreased trend in OARSI scores among the MSMP groups.

### 3.3 MSMP affected cartilage matrix metabolism in OA mice

**FIGURE 2:**
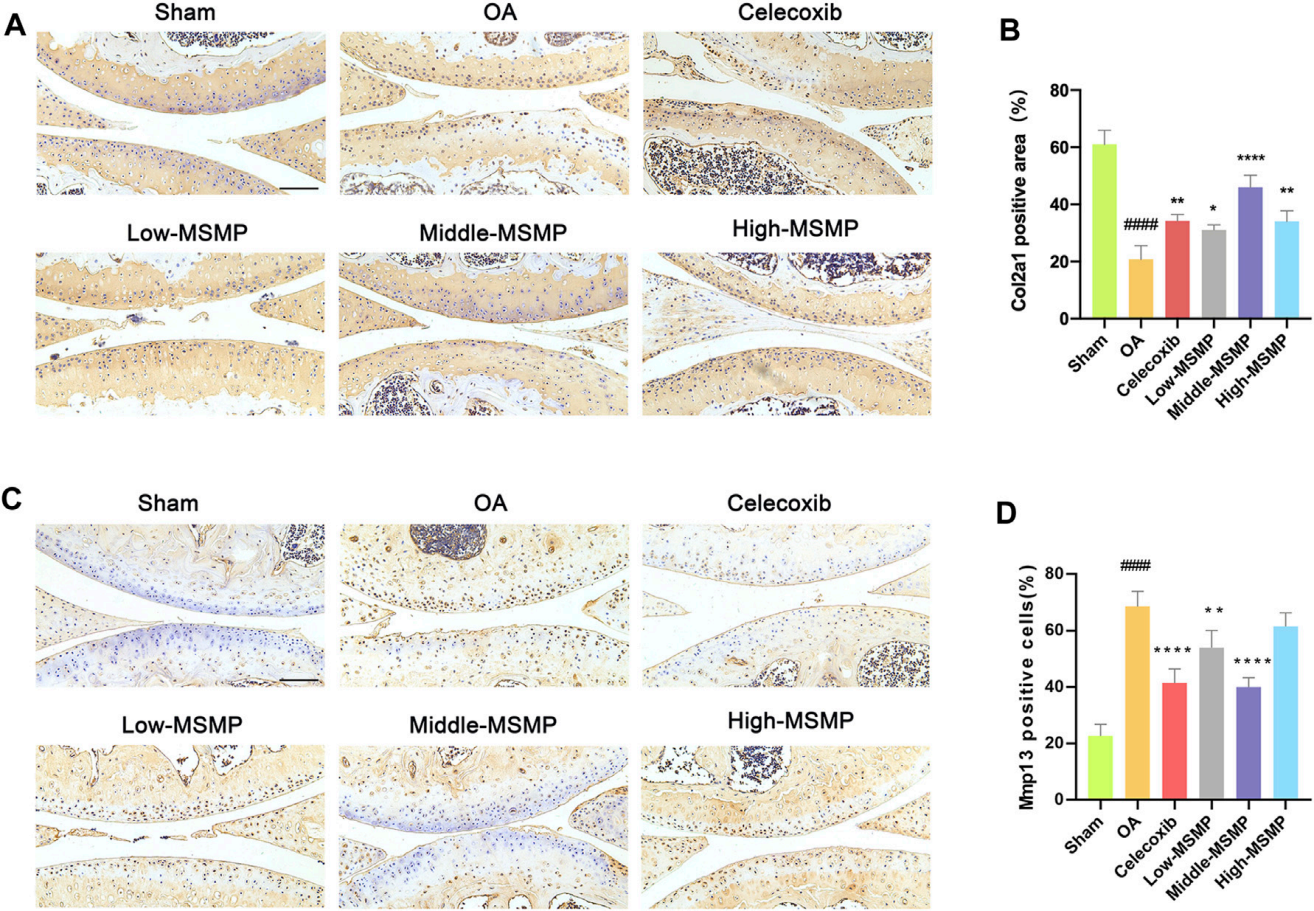
MSMP affected cartilage matrix metabolism in OA mice. **(A)** Immunohistochemical staining of Col2a1. **(B)** Statistical analysis of the positive expression for Col2a1. **(C)** Immunohistochemical staining of Mmp13. **(D)** Statistical analysis of the positive expression for Mmp13. Scale bar represents 100 μm (Date are mean ± SD, ^####^ *p* < 0.0001, versus Sham group; *p < 0.05, **p < 0.01, ****p < 0.0001, versus OA group).

Col2a1 and Mmp13 immunohistochemical staining was performed in order to investigate the effect of MSMP on cartilage matrix metabolism in OA mice. The results showed that the expression of Col2a1 in the articular cartilage in the OA group was very weak, and that the Col2a1 expression was significantly strong in the articular cartilage in the treated groups, especially in the middle-MSMP group (Figures 2A, B). The results of immunohistochemical staining of Mmp13 indicated that the expression of Mmp13 was significantly attenuated to varying degrees in mice intervened with celecoxib and MSMP compared with the mice in the OA group, accompanied with the most significant decrease in the middle-MSMP group among MSMP groups (Figures 2C, D).

### 3.4 MSMP inhibited synovial inflammation and regulated macrophage polarization in OA mice

**FIGURE 3:**
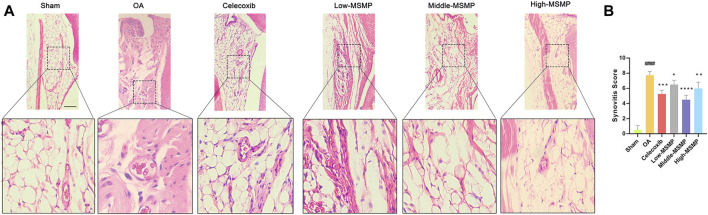
MSMP inhibited synovial inflammation in OA mice. **(A)** HE staining of the synovium in mice. **(B)** Synovitis score in mice; Scale bar represents 100 μm (Date are mean ± SD, ^####^ *p* < 0.0001, versus Sham group; *p < 0.05, **p < 0.01, ***p < 0.001, ****p < 0.0001, versus OA group).

HE staining of synovium was performed to evaluate the effect of MSMP on synovial inflammation in OA mice (Figure 3A). The degree of synovia inflammation was evaluated by synovitis score (Figure 3B). As shown in Figure 3A, the synovial tissue displayed clear borders without inflammatory infiltration, and the synovial interstitial cells had a regular structure in the sham group, whereas the cells were characterized by increased density, a disorganized structure, obvious inflammatory infiltration and multiple vascular opacities in the OA group. The valuated results of Figure 3B manifested that synovia inflammation was greatly reduced in the treated groups. The synovitis score declined most significantly in the middle-MSMP group among MSMP groups.

**FIGURE 4:**
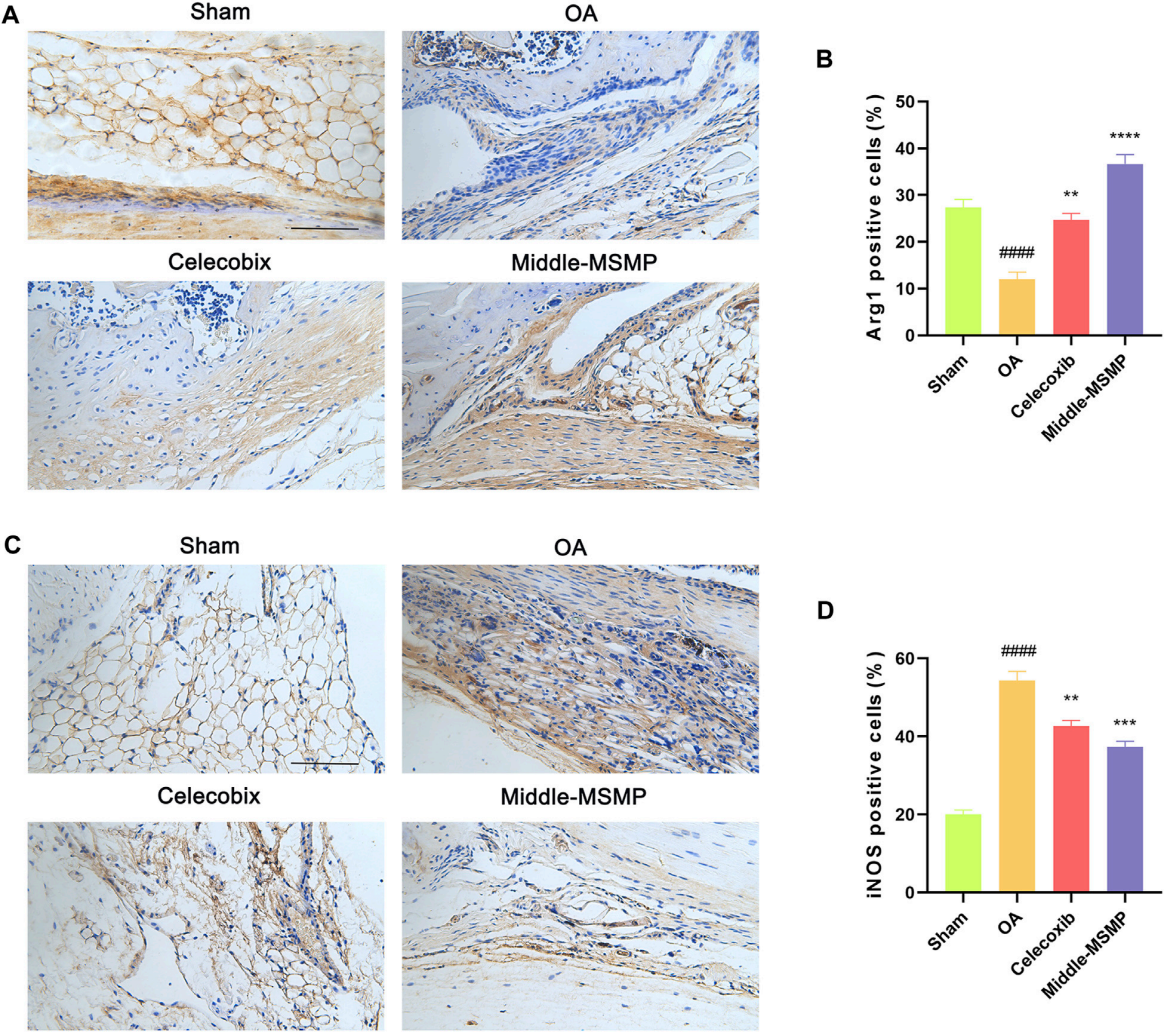
MSMP was involved in immunoregulation of synovial macrophages. **(A)** Immunohistochemical staining of Arg1. **(B)** Statistical analysis of the positive expression for Arg1 in synovial tissue. **(C)** Immunohistochemical staining of iNOS. **(D)** Statistical analysis of the positive expression for iNOS in synovial tissue. Scale bar represents 100 μm (Date are mean ± SD, ^####^ *p* < 0.0001, versus Sham group; **p < 0.01, ***p < 0.001, ****p < 0.0001, versus OA group).

Meanwhile, compared with the sham group, the OA group showed a significantly higher expression of the pro-inflammatory factor iNOS (M1 type) and a lower expression of anti-inflammatory factor Arg1 (M2 type) in the synovial tissues (Figure 4). The expression of iNOS was suppressed while the expression of Arg1 was promoted in the middle-MSMP group, suggesting that MSMP was involved in the immune regulation of synovial macrophages.

### 3.5 MSMP affected the expression levels of serum IL-6, IL-10, and TNF-α in OA mice

**FIGURE 5:**
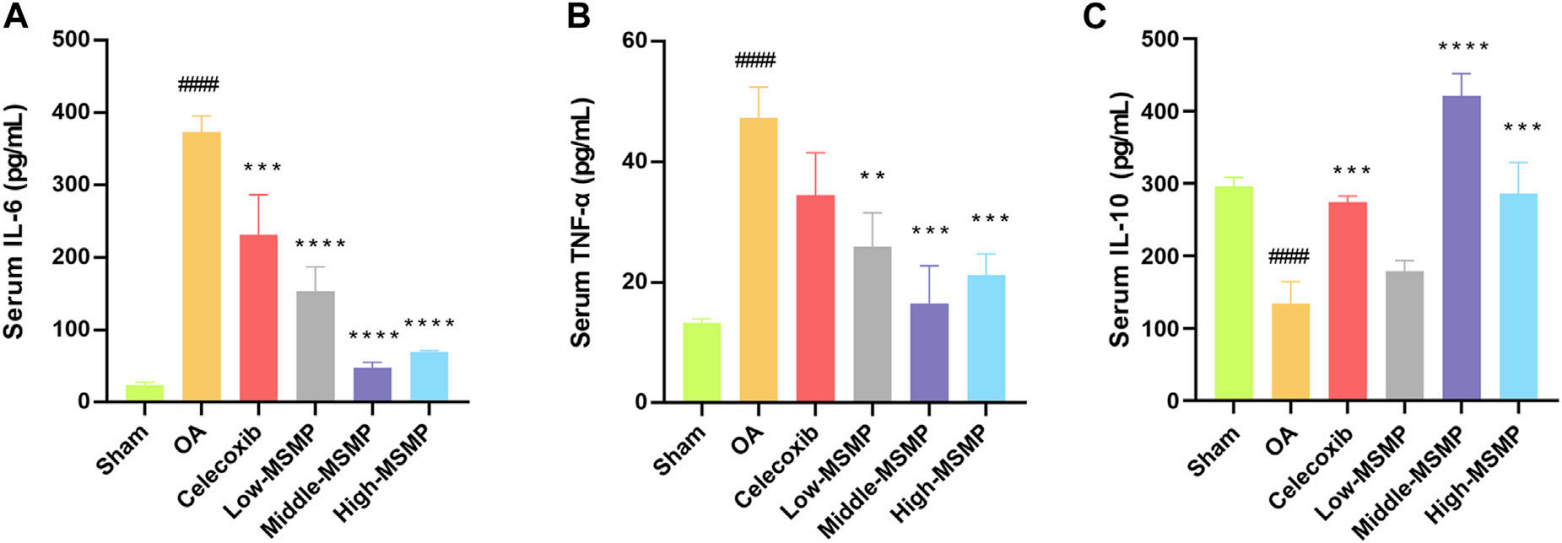
MSMP affected the expression levels of serum IL-6, IL-10 and TNF-α in OA mice. **(A)** Expression level of serum IL-6; **(B)** Expression level of serum TNF-α; **(C)** Expression level of serum IL-10 (Date are mean ± SD, ^####^ *p* < 0.0001, versus Sham group; **p < 0.01, ***p < 0.001, ****p < 0.0001, versus OA group).

The expression level of IL-6 and TNF-α was significantly increased (Figures 5A, B) while the expression level of IL-10 was dramatically decreased (Figure 5C) in the serum of the OA group compared with those in the sham group. In contrast with the OA group, the expression of inflammatory and anti-inflammatory factors in the treated groups was significantly changed after the administration of celecoxib or MSMP gavage, and the middle-MSMP group showed the optimal inhibited effect on the expression of inflammatory factors and promoted effect on the expression of anti-inflammatory factor among MSMP groups.

### 3.6 MSMP-containing serum regulated M1/M2 polarization of macrophages

**FIGURE 6:**
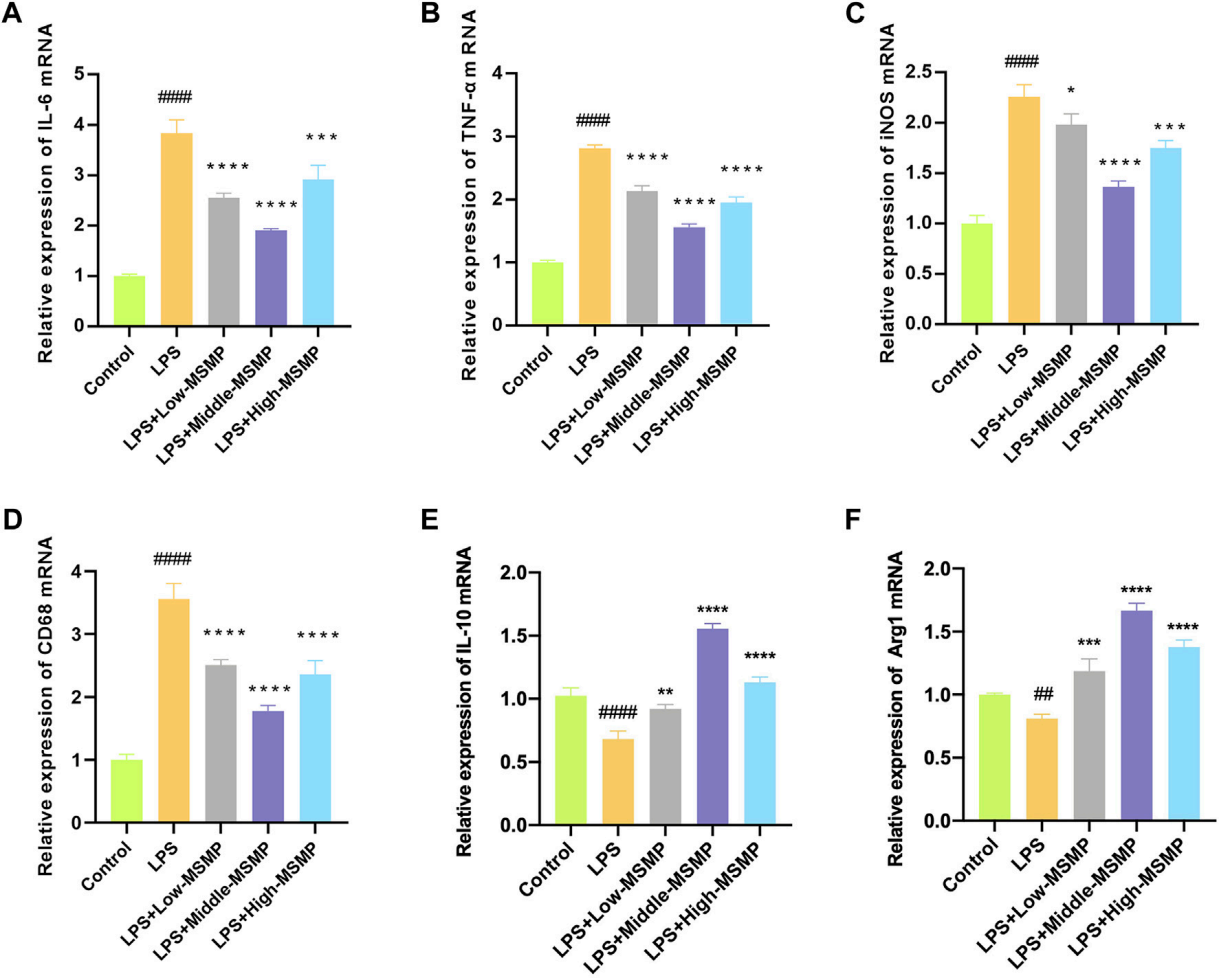
MSMP-containing serum regulated M1/M2 polarization of macrophage. **(A)** Relative mRNA expressions of IL-6; **(B)** Relative mRNA expressions of TNF-α; **(C)** Relative mRNA expressions of iNOS; **(D)** Relative mRNA expressions of CD86; **(E)** Relative mRNA expressions of IL-10; **(F)** Relative mRNA expressions of Arg1 (Date are mean ± SD, ^####^ *P* < 0.0001, versus Control group; *p < 0.05, **p < 0.01, ***p < 0.001, ****p < 0.0001, versus LPS group).

The mRNA level of pro-inflammatory or anti-inflammatory markers was tested to investigate the effect of MSMP-containing serum on macrophage polarization. In the LPS group, the expression of CD86, iNOS, TNF-α, and IL-6 was increased, while the expression of Arg1 and IL-10 was decreased, suggesting that LPS promoted the M1 polarization and inhibited the M2 polarization of macrophages (Figure 6). After intervention with MSMP-containing serum, the expression of CD86, iNOS, TNF-α, and IL-6 was decreased, while the expression of Arg1 and IL-10 was increased demonstrating that MSMP-containing serum promoted the polarization of macrophages from M1 to M2 phenotype. The most significant change was observed in the middle-MSMP group.

### 3.7 MSMP-containing serum inhibited NF-κB pathway in macrophages

**FIGURE 7:**
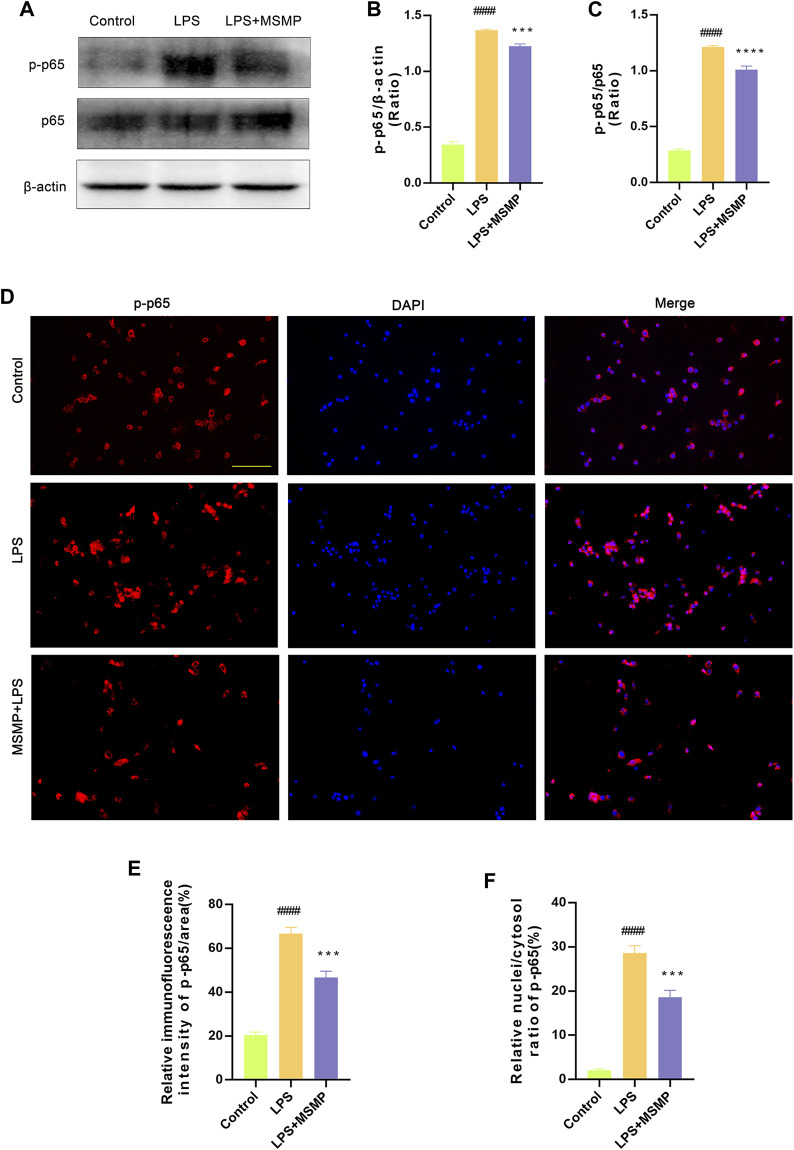
MSMP-containing serum inhibited NF-κB pathway in macrophages after LPS induction. **(A)** Western blot analysis of p-p65, p65 and β-actin; **(B)** Statistical analysis of the Western blot results for p-p65/β-actin (%); **(C)** Statistical analysis of the Western blot results for p-p65/p65 (%); Quantification of the proteins was analyzed by the ImageJ software. **(D)** The immunofluorescence assay shows the blue fluorescence for DAPI, the red fluorescence for p-p65 and finally the merge plot; **(E, F)** The expression of p-p65 was analyzed by the ImageJ software. Scale bar represents 100 μm (Date are mean ± SD, ^####^ *p* < 0.0001, versus Control group; ***p < 0.001, ****p < 0.0001, versus LPS group).

Western blotting and immunofluorescence assay were performed to explore the molecular mechanism underlying the immunoregulation of MSMP-containing serum on macrophages. The results of Western blotting and data analyses showed that the relative expression of p-p65 protein was significantly enhanced in macrophages induced with LPS compared with controls, while the middle-MSMP-containing serum partly reversed the expression of p-p65 (Figures 7A–C). The results of immunofluorescence and data analyses demonstrated that the p-p65 expression was weak in the control group, whereas the p-p65 expression was significantly enhanced and p-p65 protein partially translocated into the nucleus in the LPS group. The serum-containing group suppressed the expression and nuclear translocation of p-p65 (Figures 7D–F). Therefore, LPS could activate the NF-κB pathway, whereas MSMP-containing serum inhibited the NF-κB pathway in macrophages.

## 4 Discussion

**FIGURE 8:**
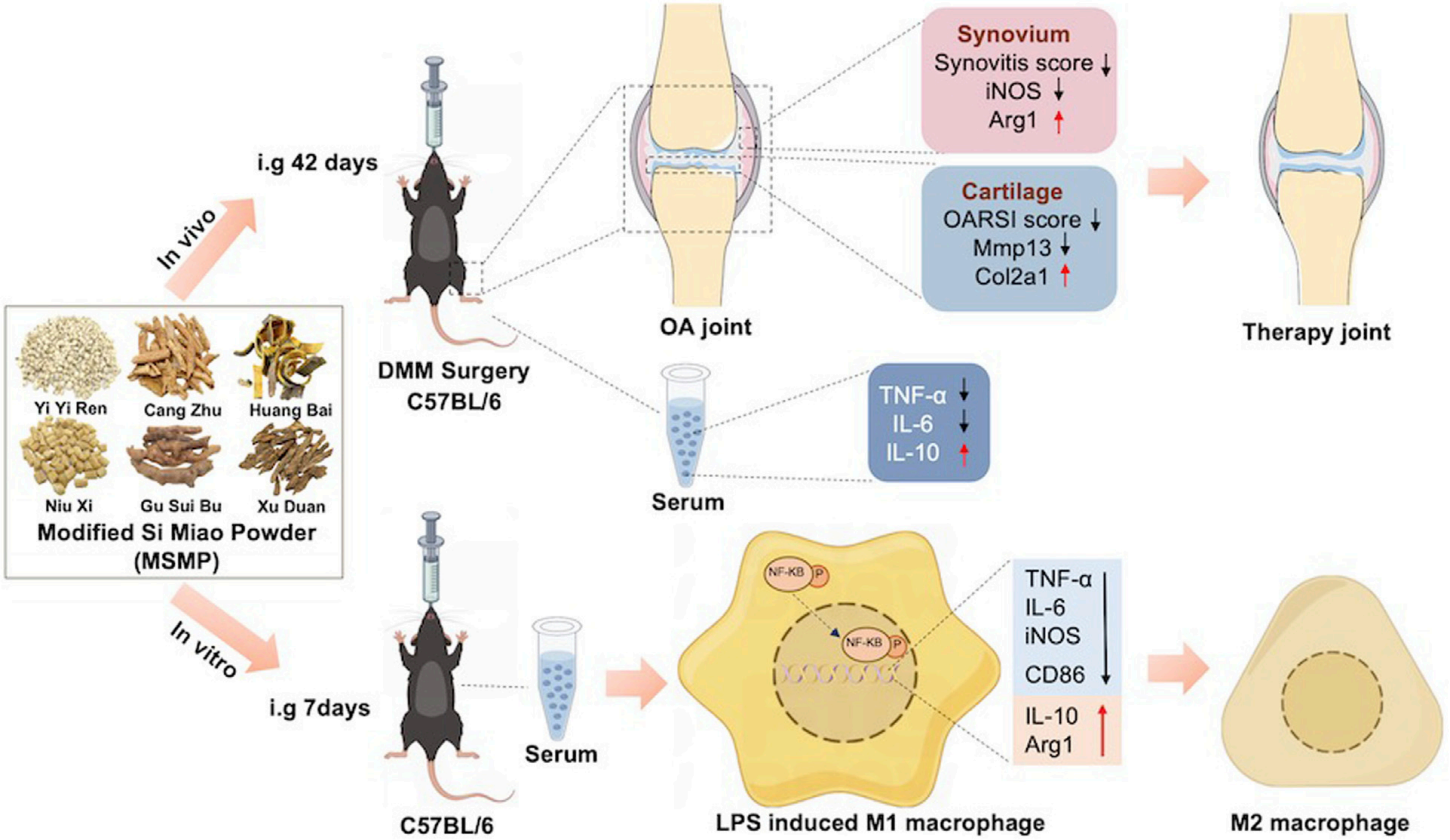
The schematic diagram shows experimental steps and therapeutic effects of MSMP on OA *in vivo* and *in vitro*. In the *in vivo* experiments, MSMP suppressed cartilage degeneration, synovial inflammation and affected the expression levels of inflammatory factors in the serum of OA mice; in the *in vitro* experiments, MSMP switched the phenotype of macrophages by inhibiting the NF-κB signalling pathway.

The present study revealed the therapeutic effects of MSMP on OA mice and the regulation of MSMP-containing serum on macrophage phenotype. As shown in Figure 8, *in vivo* studies showed that the articular cartilage degeneration was reduced and the synovial inflammation was alleviated in the mice treated with MSMP by gavage compared with OA mice. The expression of increased Col2a1 and decreased Mmp13 in articular cartilage showed that MSMP enhanced anabolism and attenuated catabolism of cartilage matrix in the treated mice. The expression of changed Arg1 and iNOS in synovium indicated that MSMP partly reversed the M1/M2 polarization imbalance of synovial macrophages in the treated mice. The increased IL-6 and TNF-αthe decreased IL-10 in serum in the treated mice implied that MSMP inhibited the inflammation. In *in vitro* studies, an inflammatory model of mouse macrophages was induced by LPS, followed by treatment with MSMP-containing serum intervention. A decrease in the expression of pro-inflammatory markers of CD86, iNOS, TNF-α and IL-6, and an increase in the expression of anti-inflammatory markers of Arg1 and IL-10 in the intervened group demonstrated that MSMP-containing serum switched M1/M2 phenotype of macrophages. Furthermore, MSMP-containing serum inhibited the NF-κB signaling pathway after LPS induction. The function of the middle MSMP was most effective for OA treatment and the middle-MSMP containing serum showed the optimal ant-inflammatory effect in macrophages, indicating that the therapeutic effect of MSMP is relatively sensitive and the concentration of the botanical drug needs to be controlled within a certain range.

OA is a chronic joint disease characterized by destruction of articular cartilage and inflammatory infiltration of synovium. The articular cartilage is a tough, tension-bearing connective tissue that covers the surface of joints, not containing blood vessels or lymphatic vessels. The synovium is a layer of loose connective tissue attached to the inside of the joint capsule, and is rich in blood vessels important for the nutrition of articular cartilage. The synovial fluid secreted by the synovium has a lubricating effect on the tissues in the joint cavity and plays a protective role in the articular cartilage (Cheleschi et al., 2022). In recent studies, the presence of synovial lesions has become an essential pathological manifestation in the onset of OA and one of the dominant factors in the development of OA (Ishibashi et al., 2020; Oo et al., 2020). The inner layer of the synovium is called the cellular layer, which consists of one to three cell layers, mainly including synovial macrophages, synovial fibroblasts and mast cells. Synovial macrophages are the main inflammatory cells involved in synovial inflammation, possessing both pro-inflammatory (M1) and anti-inflammatory (M2) phenotypes. M1-type macrophages secrete inflammatory factors such as IL-1β, IL-6, and TNF-α, which further accelerate synovial inflammation and disrupt the balance of articular cartilage matrix metabolism. M2-type macrophages secrete anti-inflammatory factors such as Arg1 and IL-10, which play a fundamental role in inhibiting inflammation (Wang et al., 2018; Liao et al., 2020). Therefore, the local inflammatory response in joint tissues can further activate M1 phenotype of macrophages and accelerate the degradation of articular cartilage. Promoting the polarization of synovial macrophages from M1 to M2 phenotype can inhibit synovial inflammation and play a protective role for articular chondrocytes (Sellam and Berenbaum, 2010; Berenbaum, 2012). In our study, we found that MSMP promoted the transition from M1 phenotype to M2 phenotype of macrophages *in vivo* and *in vitro*.

The herbal formula of MSMP in the present study included six Chinese herb. The active metabolite of Atractylodes Rhizome, atractylone, has significant anti-inflammatory and analgesic effects (Koonrungsesomboon et al., 2014). The study of Chen et al. showed that atractylone could inhibit LPS-induced NO and COX-2 expression in RAW264.7 cells (Chen et al., 2016). Qu et al. reported that the extracted atractylodes oil from Atractylodes Rhizome could effectively suppress the expression of pro-inflammatory factors such as TNF-α and IL-6 in the serum of rats with adjuvant arthritis (Qu et al., 2020). Cortex phellodendri has been demonstrated to have significant anti-inflammatory effects, with berberine and citrulline-based alkaloids as the major anti-inflammatory metabolites (Fujii et al., 2017). Yan et al. found that Huang Bai Jian Pi decoction affected PI3K/Akt/NF-κB signaling pathway and suppressed the expression of inflammatory cytokines in a TNF-α-induced diarrhea model of rats (Yan et al., 2022). Achyranthis Bidentatae Radix is widely used in Chinese orthopedics and traumatology for the treatment of OA. Weng et al. found that Achyranthis Bidentatae polysaccharides promoted chondrocyte proliferation and upregulated the expression of type II collagen (Weng et al., 2014). β-ecdysterone, as a major metabolite of Achyranthis Bidentatae Radix, suppressed inflammation and apoptosis in rat chondrocytes by preventing NF-κB signaling pathway (Zhang et al., 2014). As a commonly employed tonic in traditional medicine, Coicis Semen has a mild effect, and is also widely used in daily life for dietary therapy owing to the belief that it can improve the immunity of the body (Zeng et al., 2022). Eriodictyol and eramide were first identified from the extract of Coicis Semen. They showed significant anti-inflammatory activity in LPS-induced RAW264.7 cells (Huang et al., 2009). Asperosaponin VI, extracted from Dipsaci Radix, decreased the levels of TNF-α and IL-6 in myocardial infarction rats and collagen induced arthritis rats (Li et al., 2012; Liu et al., 2019). Drynariae Rhizoma is a common adjuvant in orthopedics. It has been indicated that total flavonoids and naringenin, which are effective metabolite extracted from Drynariae Rhizoma, could inhibit inflammation and downregulate the expression of matrix metalloproteinases in articular cartilage (Zhao et al., 2021; Pan et al., 2022).

NF-kB signaling pathway is pivotal to TLR-medicated inflammation responses in all macrophages. LPS acts as an endotoxin that is mainly recognized by TLR4 in the cell membrane of macrophages, which can promote the expression of NF-κB signaling pathway (Xiang et al., 2011; Zhao et al., 2019). NF-kB is an important nuclear transcription factor involved in the activation of various pro-inflammatory factors, including iNOS, TNF-α and IL-6 (Wang and He, 2022). In the previous studies, the activation of the NF-κB pathway promotes macrophages to release pro-inflammatory molecules and tend to serve as M1 phenotype (Lu et al., 2021). On the contrary, the inhibition of the NF-κB pathway leads to the upregulation of anti-inflammatory factors and the M2 polarization of reprogrammed macrophage, thus contributing to the prevention and treatment of some disease, such as liver inflammation and acute myocardial infarction (Li et al., 2020; Cai et al., 2022; Zhao et al., 2022). In the present study, MSMP switched M1 to M2 phenotype of macrophages in synovium of OA mice, which could inhibit synovitis and thus change the microenvironment in knee joint. The improved anti-inflammatory microenvironment might inhibit the chondrocyte death and the degradation of cartilage matrix, and thus alleviated OA progression. Meanwhile, the expression of inflammation related factors was also changed in the serum of OA mice. Furthermore, MSMP promoted the transition of macrophages from M1 to M2 phenotype by inhibiting the NF-κB pathway after LPS induction *in vitro*. The effects and molecular mechanism of MSMP on subchondral bone have yet to be further investigated.

## 5 Conclusion

In this study, we found that MSMP inhibited articular cartilage damage, balanced the cartilage matrix metabolism, slowed down synovitis, switched macrophage M1 to M2 polarization, decreased serum IL-6 and TNF-α levels and increased serum IL-10 levels in OA mice. Therefore, MSMP improved the microenvironment of knee joint and alleviated OA progression. The drug-containing serum of MSMP modulated M1-type and M2-type polarization of synovial macrophages by inhibiting the NF-κB signaling pathway. Taken together, this study provided an experimental basis for the therapeutic effect of MSMP on OA, as well as ideas for the therapeutic targets of MSMP in the treatment of OA.

## Data Availability

The original contributions presented in the study are included in the article/Supplementary Material, further inquiries can be directed to the corresponding authors.
