# Corrigendum: Modified Si Miao powder granules alleviates osteoarthritis progression by regulating M1/M2 polarization of macrophage through NF-κB signaling pathway

**DOI:** 10.3389/fphar.2024.1480427

**Published:** 2024-08-22

**Authors:** Qi He, Ding Tian, Zhiyuan Wang, Dan Zheng, Liqiang Zhi, Jianbing Ma, Jing An, Rui Zhang

**Affiliations:** ^1^ Translational Medicine Center, Honghui Hospital, Xi’an Jiaotong University, Xi’an, Shaanxi, China; ^2^ Department of Joint Surgery, Honghui Hospital, Xi’an Jiaotong University, Xi’an, Shaanxi, China; ^3^ Department of Medical Technology, Guiyang Healthcare Vocational University, Guiyang, Guizhou, China

**Keywords:** osteoarthritis, inflammation, modified Si Miao Powder, macrophage polarization, NF-κB signaling pathway

In the published article, there was an error in the **Affiliation** list. The Department of Joint Surgery and Translational Medicine Center of Honghui Hospital, Xi’an Jiaotong University are two parallel departments in the Hospital. These two departments were written as one affiliation, causing an error.

The affiliations previously stated:

“1 Department of Joint Surgery, Translational Medicine Center, Honghui Hospital, Xi’an Jiaotong University, Xi’an, Shaanxi, China

2 Department of Medical Technology, Guiyang Healthcare Vocational University, Guiyang, Guizhou, China”.

The corrected affiliation list appears below:

“1 Translational Medicine Center, Honghui Hospital, Xi’an Jiaotong University, Xi’an, Shaanxi, China.

2 Department of Joint Surgery, Honghui Hospital, Xi’an Jiaotong University, Xi’an, Shaanxi, China.

3 Department of Medical Technology, Guiyang Healthcare Vocational University, Guiyang, Guizhou, China.”

The authors apologize for this error and state that this does not change the scientific conclusions of the article in any way. The original article has been updated.

