# Supplementary material for: Modified Si Miao Powder granules alleviates osteoarthritis progression by regulating M1/M2 polarization of macrophage through NF-κB signaling pathway
Source: Front Pharmacol. 2024 Jun 21;15:1361561. doi: 10.3389/fphar.2024.1361561 (PMC11224909; doi:10.3389/fphar.2024.1361561)
Supplement: Supplementary file 1 [file Table1.docx]

Supplementary Table 1 Main chemical compositions of MSMP identified by HPLC-MS/MS.

| NO. | Name | Formula | Class | Error (ppm) | RT [min] | [M ± H] | M/S | Area: raw (F2) |
| --- | --- | --- | --- | --- | --- | --- | --- | --- |
| 1 | Berberine | C20 H17 N O4 | Protoberberine alkaloids and derivatives | -2.97 | 7.9 | [M+H]+1 | 336.12200 | 3.4357E+11 |
| 2 | 4-Ethoxybenzaldehyde | C9 H10 O2 | Phenol ethers | -3.13 | 5.835 | [2M+ACN+H]+1 | 342.16904 | 17799946360 |
| 3 | Ethylmorphine | C19 H23 N O3 | Morphinans | -3.09 | 6.238 | [M+H]+1 | 314.17410 | 7201892231 |
| 4 | Betaine | C5 H11 N O2 | Carboxylic acids and derivatives | -0.72 | 0.848 | [M+H]+1 | 118.08620 | 6828908258 |
| 5 | Mexiletine | C11 H17 N O | Phenol ethers | -1.62 | 1.518 | [M+H]+1 | 180.13800 | 5450525692 |
| 6 | N-(4-Hydroxy-3-nitrophenyl)  -2-[(1-methyl-2,4,6  -trioxohexahydro-5  -pyrimidinyl)carbonyl]  hydrazinecarboxamide | C13 H12 N6 O8 | Phenols | -1.82 | 0.811 | [M+H]+1 | 381.07825 | 4946323598 |
| 7 | 3-Chlor-6  -hydrazinylpyridazin | C4 H5 Cl N4 | Diazines | 3.35 | 6.168 | [M+H+MeOH]  +1 | 177.05424 | 4870010859 |
| 8 | Choline | C5 H13 N O | Organonitrogen compounds | 1.45 | 0.798 | [M+H]+1 | 104.10713 | 3819863899 |
| 9 | (3R,5R)-1,3,5-Trihydroxy  -4-{[(2E)-3-(4-hydroxy  -3-methoxyphenyl)-2  -propenoyl]oxy}  cyclohexanecarboxylic acid | C17 H20 O9 | Organooxygen compounds | -2.74 | 6.173 | [M+H]+1 | 369.11700 | 3520683933 |
| 10 | 1,9-Pyrazoloanthrone | C14 H8 N2 O | Anthracenes | -2.57 | 9.841 | [M+H]+1 | 221.07037 | 3153159948 |
| 11 | Cinnamic acid | C9 H8 O2 | Cinnamic acids and derivatives | -3.63 | 7.462 | [2M+ACN+H]  +1 | 338.13760 | 3080856011 |
| 12 | L-Pyroglutamic acid | C5 H7 N O3 | Carboxylic acids and derivatives | -1.42 | 1.185 | [M+H]+1 | 130.04970 | 2818447791 |
| 13 | Demethyleneberberine | C19 H17 N O4 | Protoberberine alkaloids and derivatives | -2.79 | 6.885 | [M+H]+1 | 342.1221 | 2811605098 |
| 14 | 4-Methoxybenzyl 2-methyl  -4-(6-methyl-4-oxo-4H  -chromen-3-yl)-5-oxo-  1,4,5,6,7,8-hexahydro-3  -quinolinecarboxylate | C29 H27 N O6 | Benzopyrans | -2.17 | 9.111 | [M+H]+1 | 486.19006 | 2645778946 |
| 15 | Naringenin | C15 H12 O5 | Flavonoids | -3.12 | 7.101 | [M+H]+1 | 237.0748 | 2256219303 |
| 16 | (3R,5R)-1,3,5-Trihydroxy  -4-{[(2E)-3-(4-hydroxy  -3-methoxyphenyl)-2  -propenoyl]oxy}  cyclohexanecarboxylic acid | C17 H20 O9 | Organooxygen compounds | -3.48 | 6.158 | [M-H]-1 | 367.10205 | 14367523194 |
| 17 | Methyl chlorogenate | C17 H20 O9 | Organooxygen compounds | -3.85 | 5.439 | [M-H]-1 | 367.10202 | 8116304036 |
| 18 | Naringin | C27 H32 O14 | Flavonoids | -3.28 | 7.09 | [M-H]-1 | 579.16998 | 7408236158 |
| 19 | N-{[(3R,6S,9R,12S,15R,18S)  -9-(4-Aminobutyl)-15  -benzyl-6-(1-hydroxyethyl)  -12-(1H-indol-3-ylmethyl)  -5,8,11,14,17-pentaoxo-18  -(D-phenylalanylamino)  -1-thia-4,7,10,13,16  -pentaazacyclononadecan  -3-yl]carbonyl}-L-threonine | C49 H64 N10 O11 S | Carboxylic acids and derivatives | 0.61 | 10.024 | [M-2H]-2 | 499.21483 | 4281249966 |
| 20 | Cryptochlorogenic acid | C16 H18 O9 | Organooxygen compounds | -3.34 | 4.469 | [M-H]-1 | 353.08658 | 3891838785 |
| 21 | Caffeic acid 3-glucoside | C15 H18 O9 | Organooxygen compounds | -3.49 | 4.515 | [M-H]-1 | 341.08661 | 3815255093 |
| 22 | Limonin | C26 H30 O8 | Prenol lipids | -3.62 | 10.937 | [M+FA-H]  -1 | 515.19055 | 3424363742 |
| 23 | 1-Caffeoylquinic acid | C16 H18 O9 | Organooxygen compounds | -3.19 | 5.255 | [M-H]-1 | 353.08655 | 3353268887 |
| 24 | Eriocitrin | C27 H32 O15 | Flavonoids | -2.76 | 6.625 | [M-H]-1 | 595.16516 | 3106801588 |
| 25 | Sweroside | C16 H22 O9 | Organooxygen compounds | -3.62 | 5.835 | [M+FA-H]  -1 | 403.12329 | 3021736913 |
| 26 | Sucrose | C12 H22 O11 | Organooxygen compounds | -3.86 | 0.798 | [M-CI]-1 | 377.08432 | 2746666843 |
| 27 | sinapoylglucose | C17 H22 O10 | Cinnamic acids and derivatives | -2.83 | 6.442 | [M-H]-1 | 385.11292 | 2408479197 |
| 28 | Picloxydine | C20 H24 Cl2 N10 | Benzene and substituted derivatives | 1.06 | 0.821 | [M-H+HAc]  -1 | 533.17059 | 1967033123 |
| 29 | Isochlorogenic acid B | C25 H24 O12 | Organooxygen compounds | -3.3 | 7.315 | [M-H]-1 | 515.11780 | 1953956117 |
| 30 | (15Z)-9,12,13-Trihydroxy-15  -octadecenoic acid | C18 H34 O5 | Fatty Acyls | -3.89 | 9.942 | [M-H]-1 | 329.23206 | 1910795179 |
