# Supplementary material for: Modified Si Miao Powder granules alleviates osteoarthritis progression by regulating M1/M2 polarization of macrophage through NF-κB signaling pathway
Source: Front Pharmacol. 2024 Jun 21;15:1361561. doi: 10.3389/fphar.2024.1361561 (PMC11224909; doi:10.3389/fphar.2024.1361561)
Supplement: Supplementary file 2 [file DataSheet1.doc]

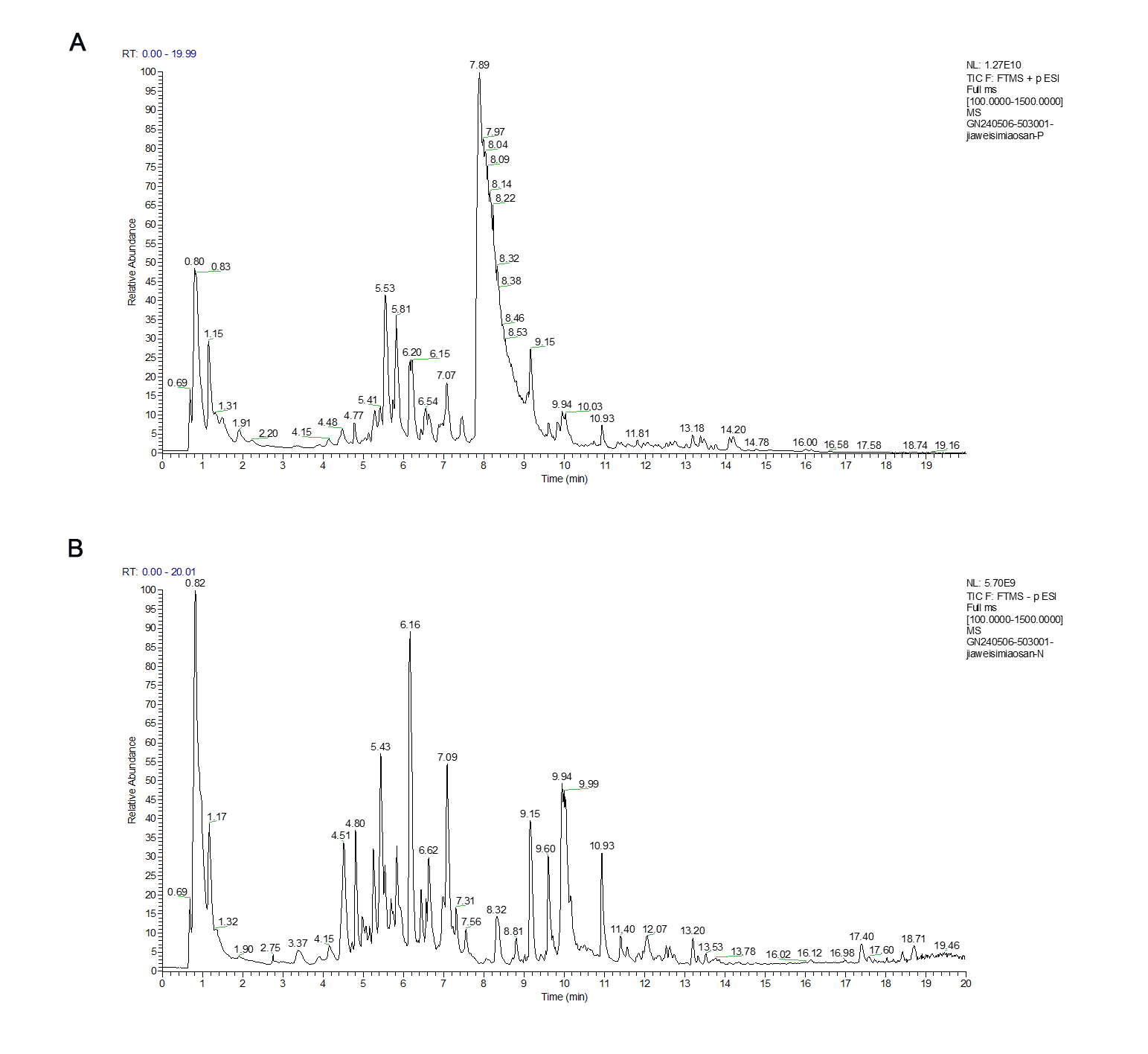


Supplementary Figure 1 Basic Particle Flow Chromatogram of MSMP (Chinese name: jiaweisimiaosuan) in positive and negative ion modes analyzed using UHPLC-MS/MS.
