# Supplementary figures and images for: Modified Si Miao Powder granules alleviates osteoarthritis progression by regulating M1/M2 polarization of macrophage through NF-κB signaling pathway
Source: Front Pharmacol. 2024 Jun 21;15:1361561. doi: 10.3389/fphar.2024.1361561 (PMC11224909; doi:10.3389/fphar.2024.1361561)

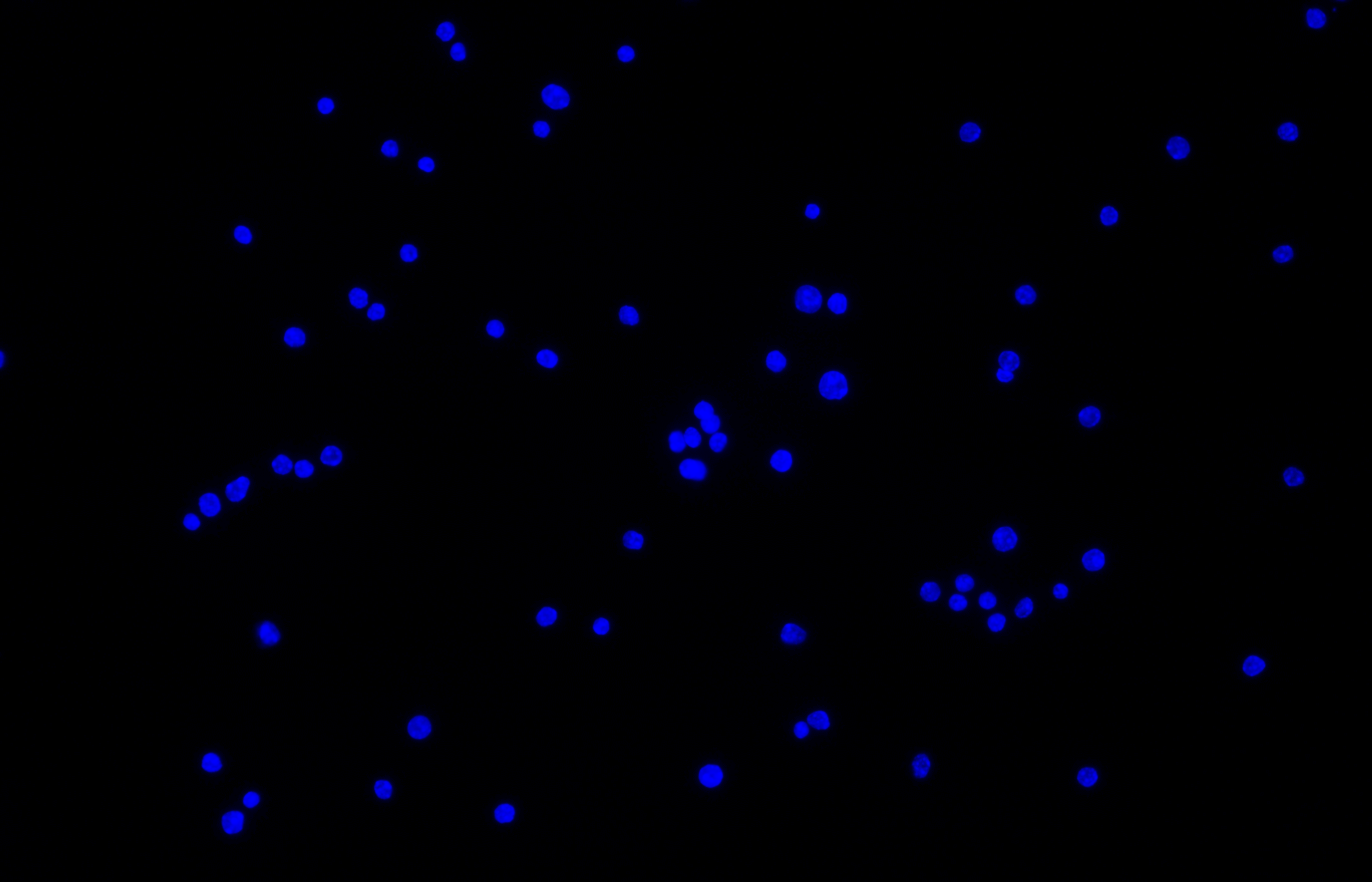

Supplement: Supplementary file 3 [file DataSheet2.zip › raw data/figure 7/IF/Control DAPI.tif]

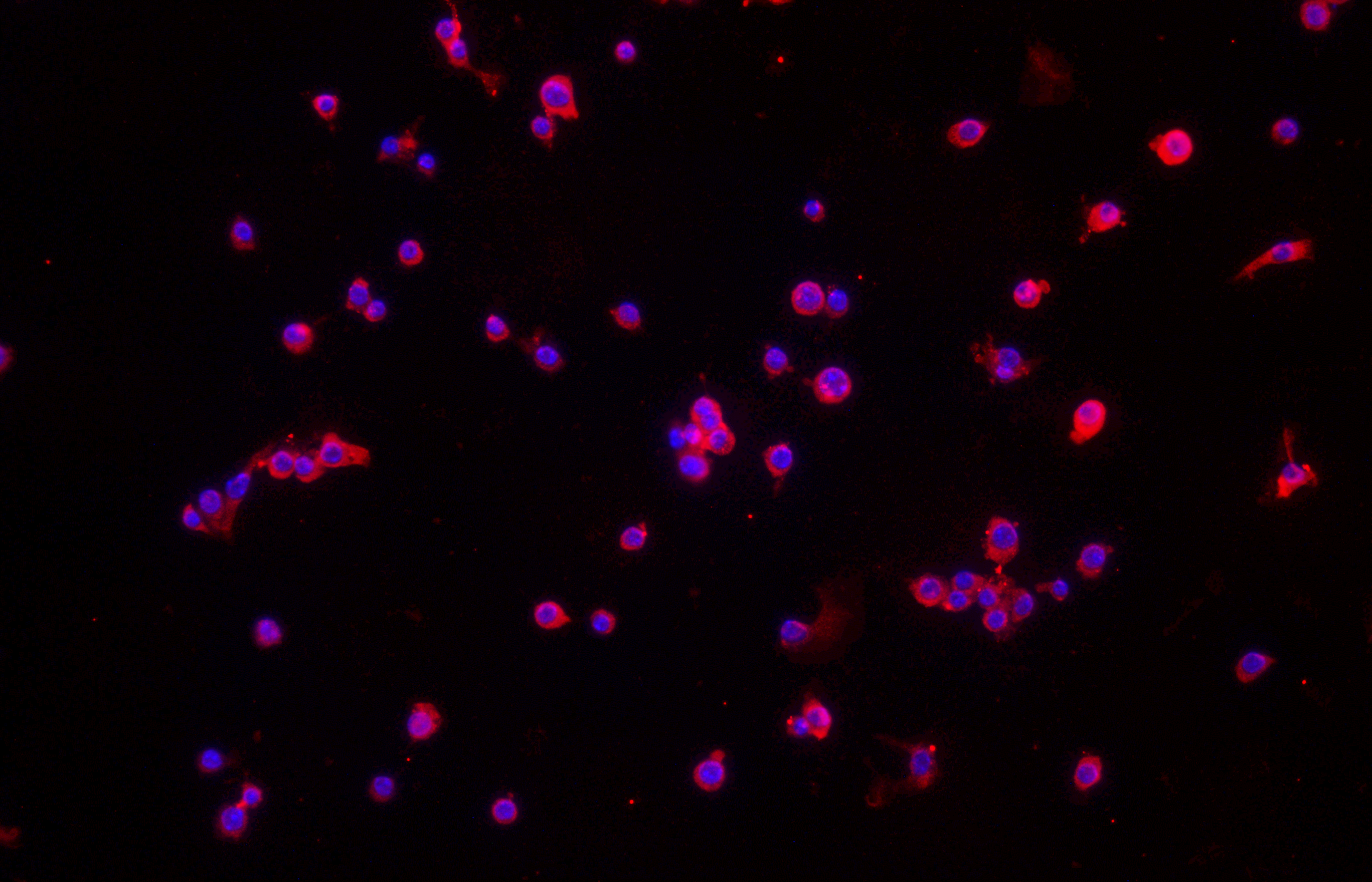

Supplement: Supplementary file 3 [file DataSheet2.zip › raw data/figure 7/IF/Control p-p65 +DAPI merge.tif]

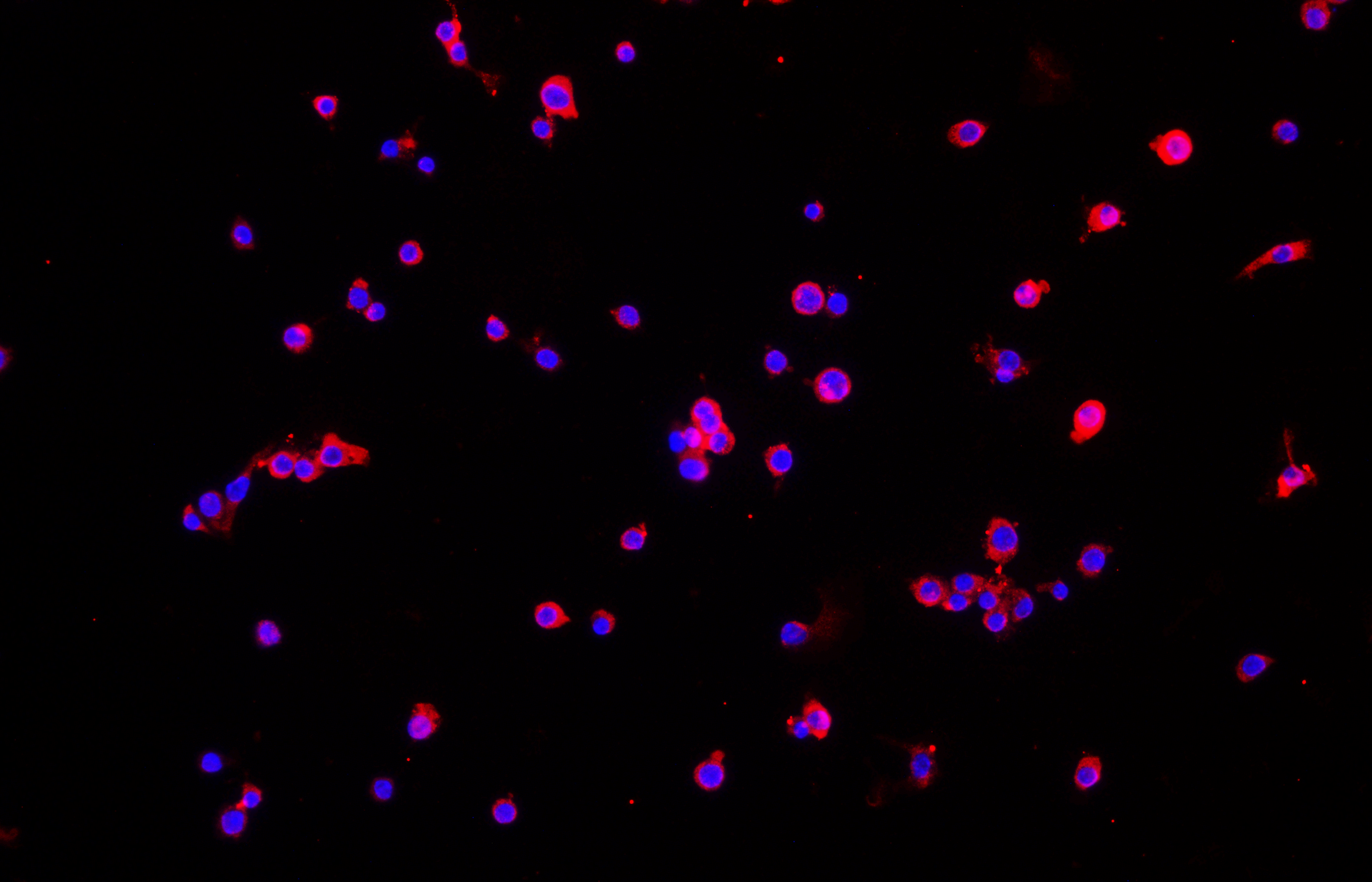

Supplement: Supplementary file 3 [file DataSheet2.zip › raw data/figure 7/IF/Control p-p65.tif]

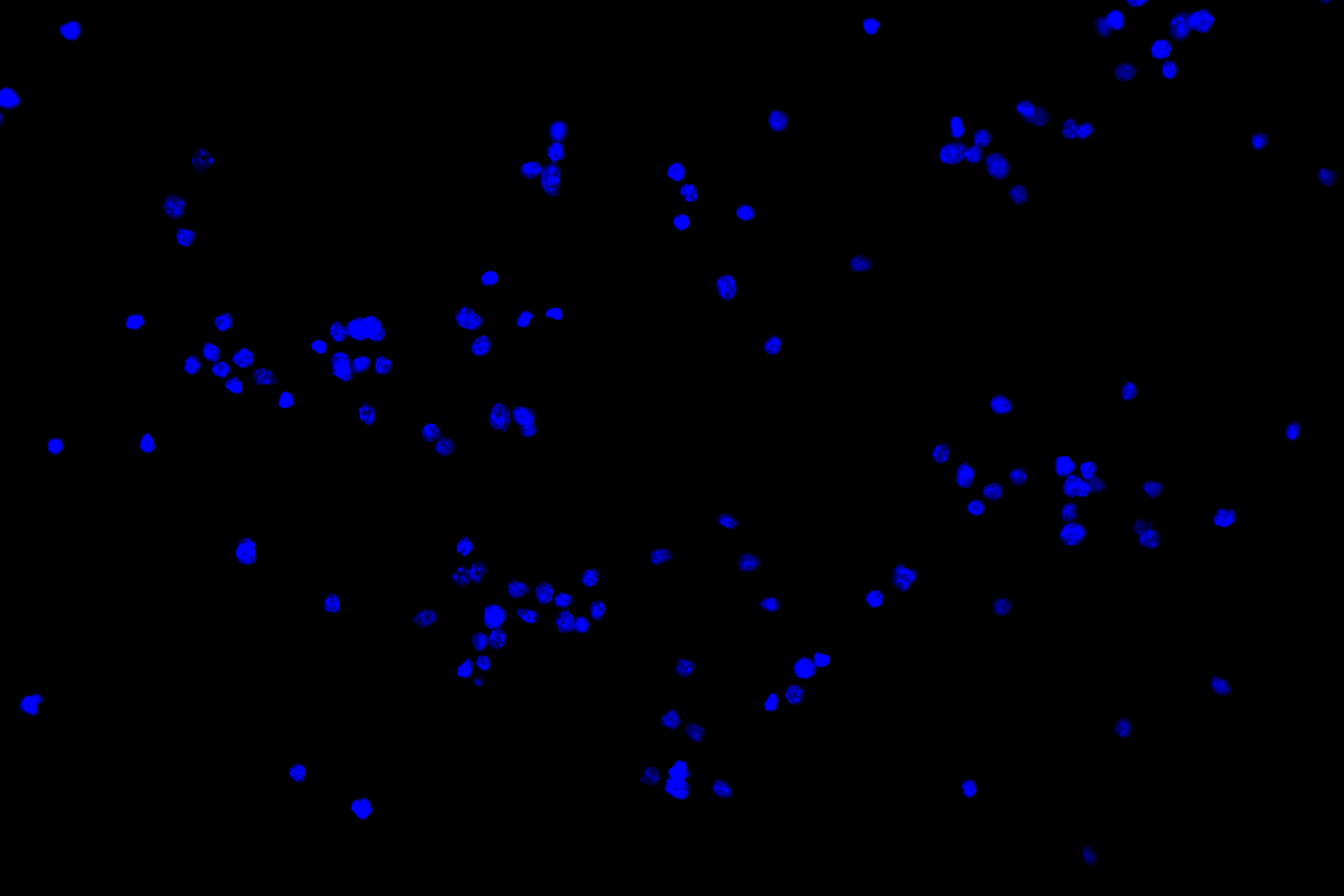

Supplement: Supplementary file 3 [file DataSheet2.zip › raw data/figure 7/IF/LPS DAPI.tif]

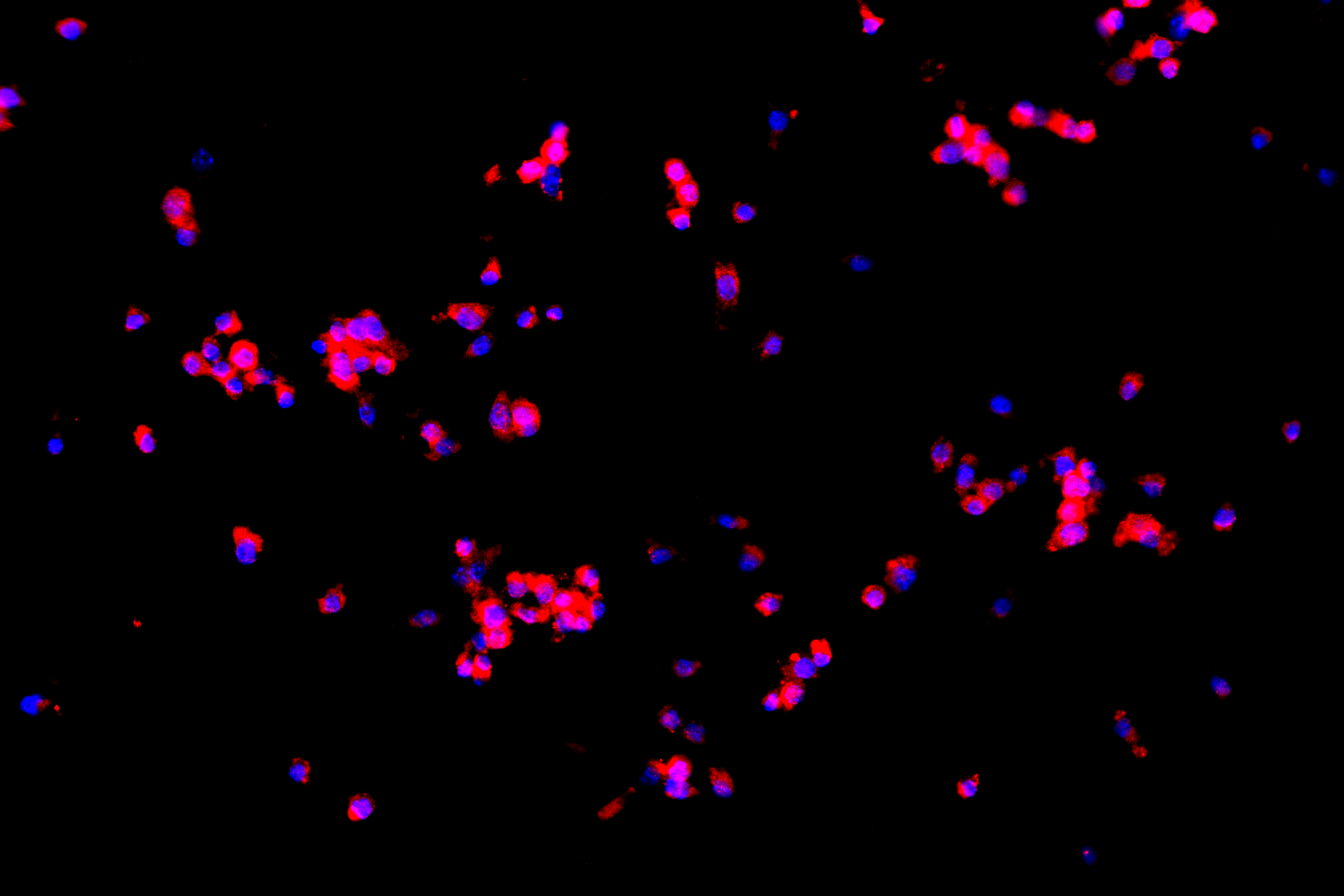

Supplement: Supplementary file 3 [file DataSheet2.zip › raw data/figure 7/IF/LPS p-p65+DAPI merge.tif]

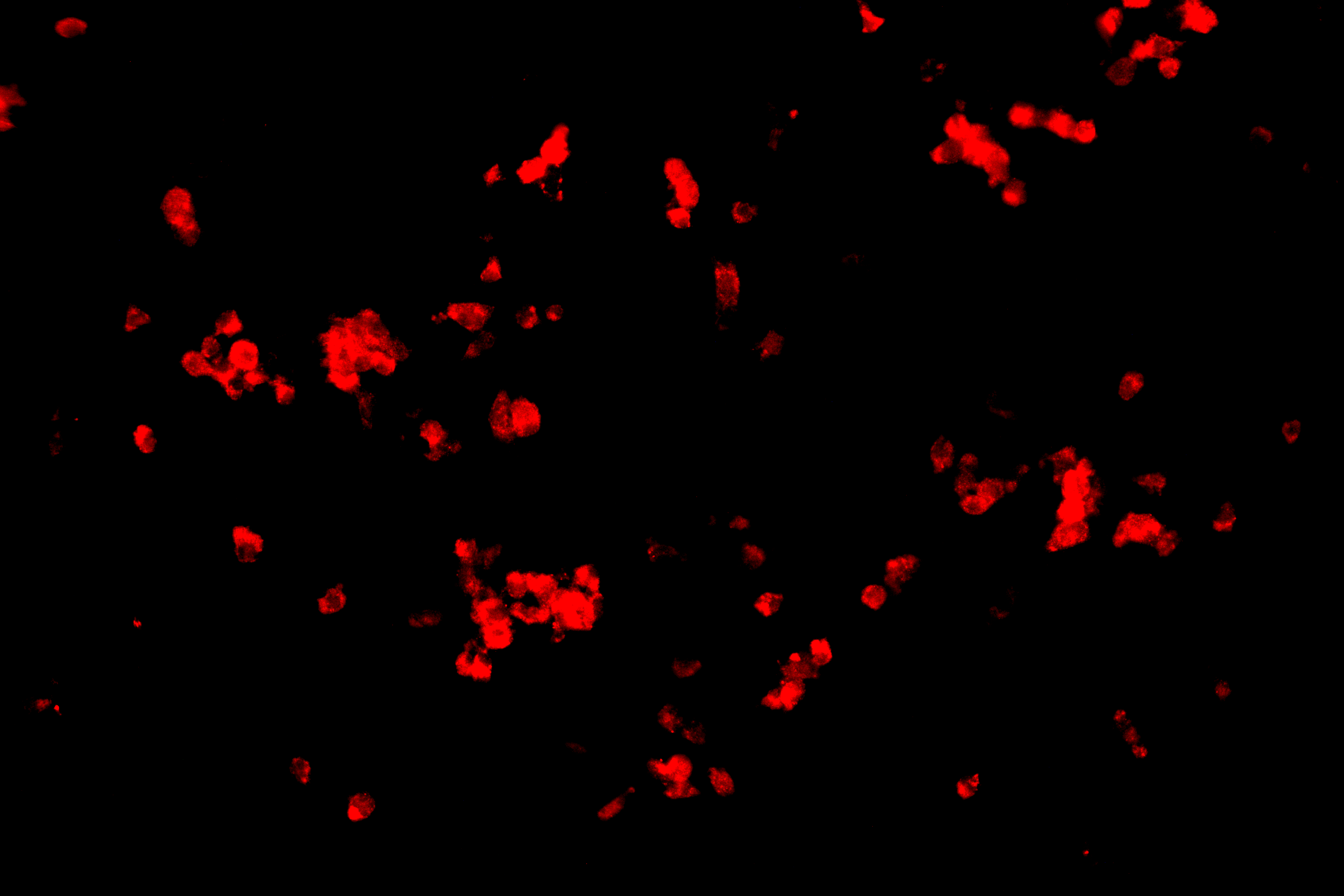

Supplement: Supplementary file 3 [file DataSheet2.zip › raw data/figure 7/IF/LPS p-p65.tif]

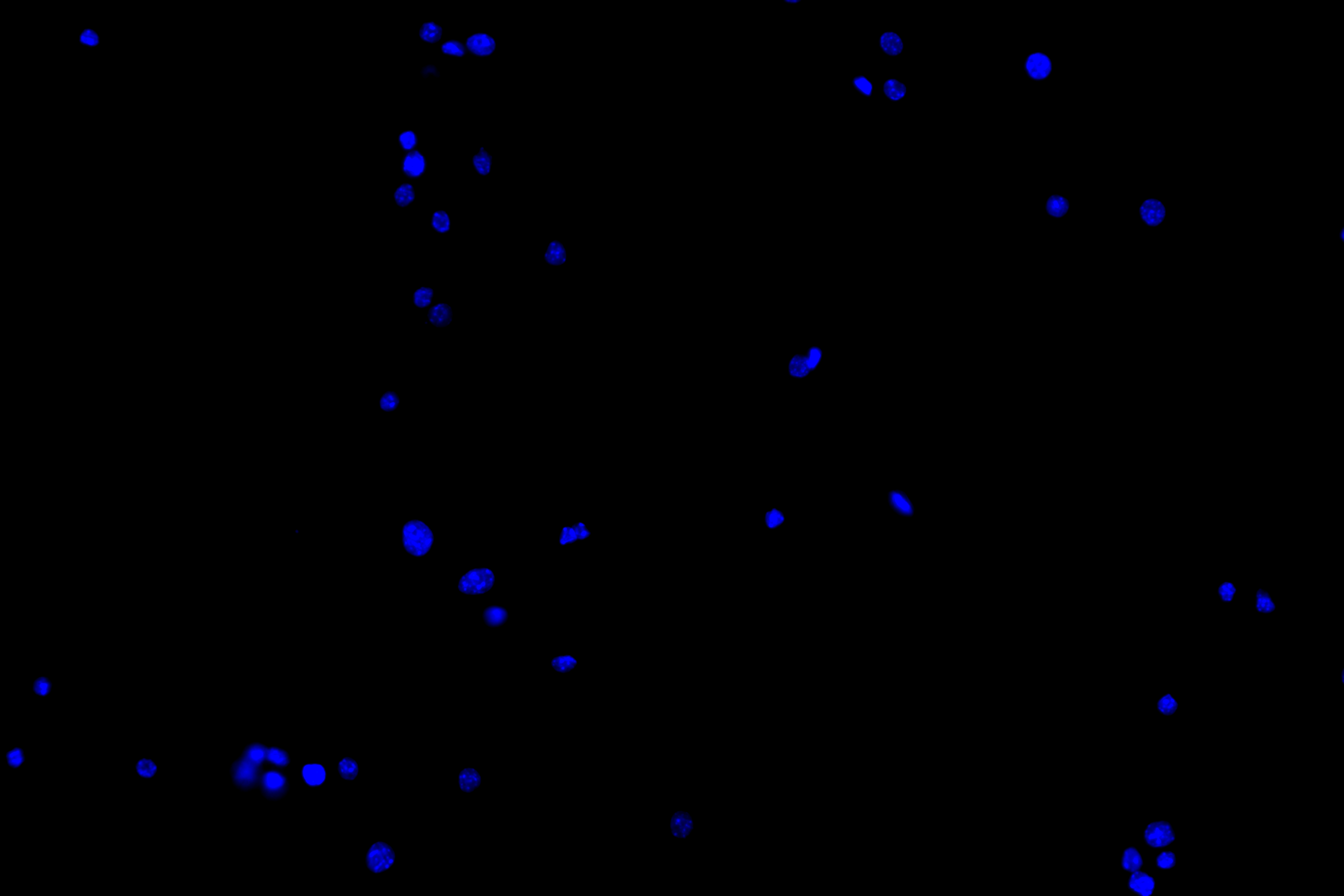

Supplement: Supplementary file 3 [file DataSheet2.zip › raw data/figure 7/IF/MSMP+LPS DAPI.tif]

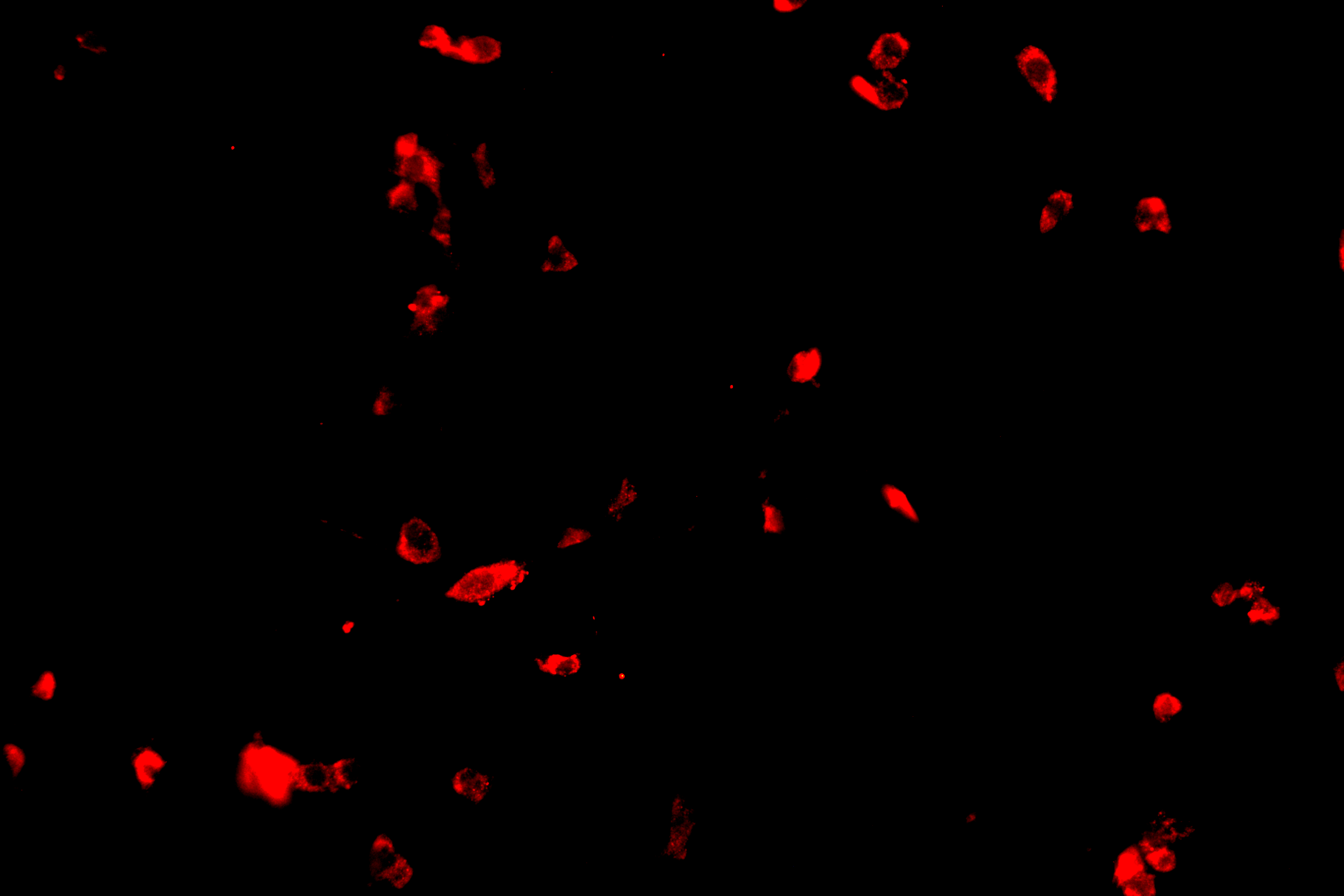

Supplement: Supplementary file 3 [file DataSheet2.zip › raw data/figure 7/IF/MSMP+LPS p-p65.tif]

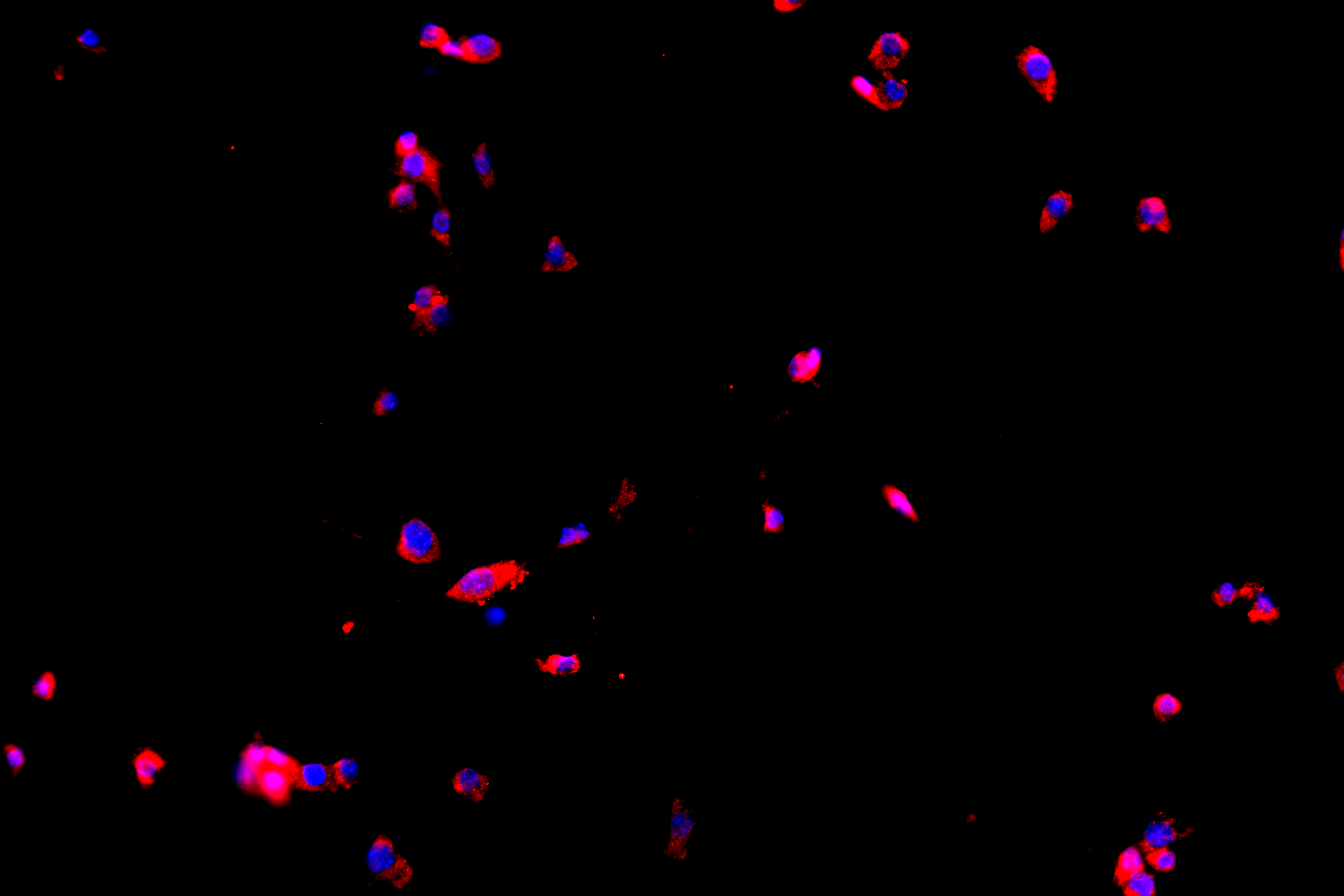

Supplement: Supplementary file 3 [file DataSheet2.zip › raw data/figure 7/IF/MSMP+LPSp-p65+DAPI merge.tif]

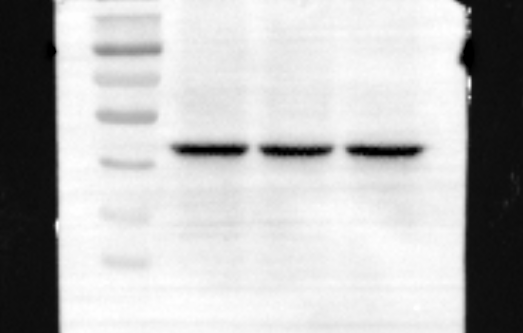

Supplement: Supplementary file 3 [file DataSheet2.zip › raw data/figure 7/WB/actin merge.tif]

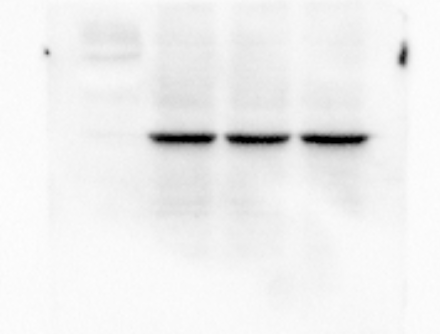

Supplement: Supplementary file 3 [file DataSheet2.zip › raw data/figure 7/WB/actin.tif]

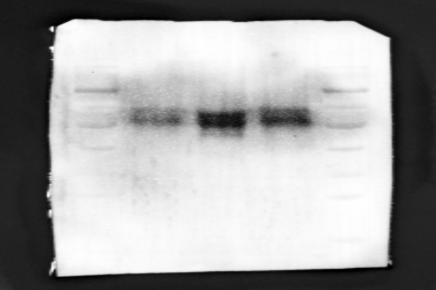

Supplement: Supplementary file 3 [file DataSheet2.zip › raw data/figure 7/WB/p-p65 merge.tif]

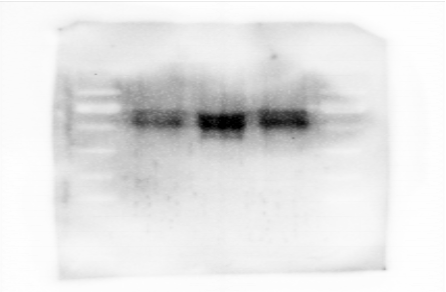

Supplement: Supplementary file 3 [file DataSheet2.zip › raw data/figure 7/WB/p-p65.tif]

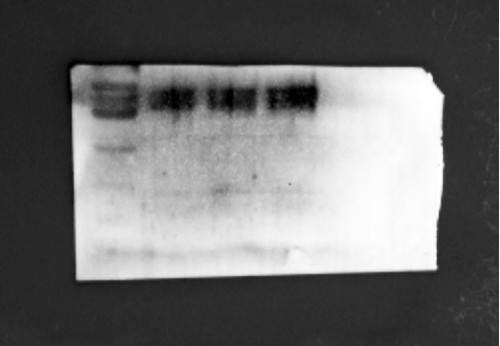

Supplement: Supplementary file 3 [file DataSheet2.zip › raw data/figure 7/WB/p65 merge.tif]

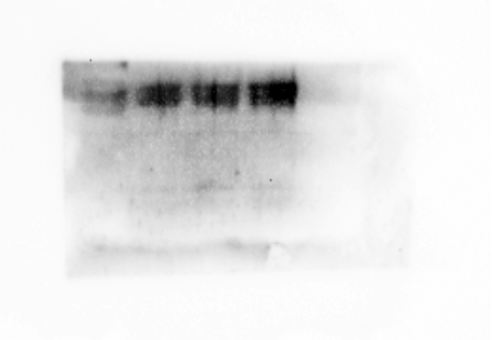

Supplement: Supplementary file 3 [file DataSheet2.zip › raw data/figure 7/WB/p65.tif]
